# Prediction of Disordered Regions and Their Roles in the Anti-Pathogenic and Immunomodulatory Functions of Butyrophilins

**DOI:** 10.3390/molecules23020328

**Published:** 2018-02-04

**Authors:** Elrashdy M. Redwan, Ahmed M. Al-Hejin, Hussein A. Almehdar, Abdelrahman M. Elsaway, Vladimir N. Uversky

**Affiliations:** 1 Department of Biological Science, Faculty of Science, King Abdulaziz University, Jeddah, PO Box 80203, Jeddah 21589, Saudi Arabia; aalhejin@kau.edu.sa (A.M.A.-H.); dralmehdar@hotmail.com (H.A.A.); 2 Therapeutic and Protective Proteins Laboratory, Protein Research Department, Genetic Engineering and Biotechnology Research Institute GEBRI, City for Scientific Research and Technology Applications, New Borg EL Arab 21934, Alexandria, Egypt; 3 Microbiology Department, Faculty of Medicine, Al-Azhar University, Cairo 11651, Egypt; elsawyeg@gmail.com; 4 Department of Molecular Medicine and USF Health Byrd Alzheimer’s Research Institute, Morsani College of Medicine, University of South Florida, Tampa, FL 33612, USA; 5 Laboratory of New Methods in Biology, Institute for Biological Instrumentation, Russian Academy of Sciences, Pushchino 142290, Moscow Region, Russia

**Keywords:** butyrophilin, structure, function, immune response, antigen presentation, intrinsic disorder

## Abstract

Butyrophilins (BTNs) are a group of the moonlighting proteins, some members of which are secreted in milk. They constitute a large family of structurally similar type 1 transmembrane proteins from the immunoglobulin superfamily. Although the founding member of this family is related to lactation, participating in the secretion, formation and stabilization of milk fat globules, it may also have a cell surface receptor function. Generally, the BTN family members are known to modulate co-stimulatory responses, T cell selection, differentiation, and cell fate determination. Polymorphism of these genes was shown to be associated with the pathology of several human diseases. Despite their biological significance, structural information on human butyrophilins is rather limited. Based on their remarkable multifunctionality, butyrophilins seem to belong to the category of moonlighting proteins, which are known to contain intrinsically disordered protein regions (IDPRs). However, the disorder status of human BTNs was not systematically investigated as of yet. The goal of this study is to fill this gap and to evaluate peculiarities of intrinsic disorder predisposition of the members of human BTN family, and to find if they have IDPRs that can be attributed to the multifunctionality of these important proteins.

## 1. Introduction

Butyrophilins (BTNs) are transmembrane glycoproteins found in the milk fat globules (MFGs) [1,2]. They constitute a large family of structurally similar type 1 transmembrane proteins belonging to the immunoglobulin superfamily, which includes adhesion molecules, receptors, and molecules of the immune system [3]. In humans, the family of *BTN* genes encodes 14 proteins that include seven BTNs grouped into three subfamilies (where the BTN1 subfamily is represented by BTN1A1, which is considered a founding member of the BTN family, the subfamily BTN2 is formed by BTN2A1, BTN2A2, and BTN2A3, whereas BTN3A1, BTN3A2, and BTN3A3 constitute the BTN3 subfamily), five BTN-like (BTNL) proteins (BTNL2, BTNL3, BTNL8, BTNL9, and BTNL10), an erythroid membrane-associated protein (ERMAP), and a myelin-oligodendrocyte glycoprotein (MOG) [4]. Two more family members, selection and upkeep of intraepithelial T cells-like protein (SKINTL) and butyrophilin-related protein 1 (BUTR1) are products of the corresponding pseudogenes [4,5]. Interestingly, only 11 BTN-related genes were identified in mouse genome [4,5,6]. Human *BTN* genes are distributed within the juxta-telomeric region of the major histocompatibility complex class I (MHCI), which contains a single copy of the *BTN1A1* gene and the *BTN2* and *BTN3* genes that have undergone tandem duplication resulting in three copies of each type of gene, giving rise to *BTN2A1*, *BTN2A2*, and *BTN2A3*, and *BTN3A1*, *BTN3A2*, and *BTN3A3* genes, respectively [5,7]. It is likely that this diversification is a relatively recent evolutionary event caused by the shuffling of exons between the two ancestral gene families [5,7].

BTNs are expressed primarily by the secretory and intestinal epithelium. Being the major protein component of milk fat droplets, they are necessary for the droplet secretion. In fact, BTN1A1 is known to regulate the secretion of milk lipid droplets, whereas BTN2A2 plays a role in lipid, fatty acids, and sterol metabolism. Additionally, BTNs can regulate T cell activation and proliferation, and have been reported to bind to xanthine-oxidase and form a complex with other proteins on the cytoplasmic part of the fat globule [8].

The human *BTN1A1* gene (or *Btn1a1* gene in mouse) is highly expressed in the secretory epithelium of the mammary gland during lactation [9,10,11]. The homologues of this gene (such as *BTNL2* and *ERMAP*) are predominantly expressed in skeletal muscle [12] and in the intestine and erythroid cells [13], respectively. On the other hand, other members of the BTN family, such as *BTN2A1*, *BTN2A2*, *BTN3A1*, *BTN3A2*, and *BTN3A3*, are characterized by the abundant expression in many tissues [14,15], suggesting that the structural domains of BTN proteins may have both universal and tissue-specific functions [9].

The goal of this article is to apply the strategy we developed earlier for the analysis of functional intrinsic disorder in milk proteins [16,17,18,19,20] in order to gain some knowledge on the potential roles of intrinsically disordered protein regions (IDPRs) in the structure and functionality of the members of the BTN family.

## 2. Results

**Figure 1:**
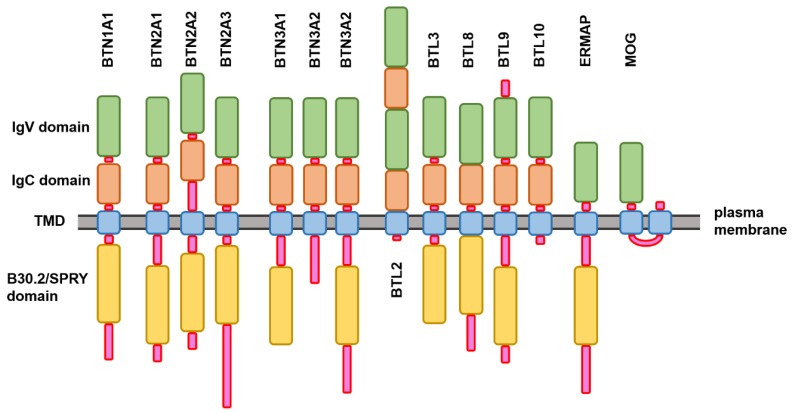
Schematic representation of the modular domain structure of the members of human butyrophilin family. Positions of various functional domains of these proteins relative to the plasma membrane are shown.

Being modular membrane proteins, members of the BTN family are characterized by similar domain organization, with classical butyrophilin consisting of three domains: two extracellular Ig-like domains, IgV and IgC, one transmembrane domain (TMD), and a cytoplasmic B30.2/SPRY domain [5]. One should remember though that not all BTN family members are made equal, and the classical domain organization is not strictly conserved, with MOG, Skint9 (not present in human), and ERMAP missing correspondingly the B30.2, IgV, and IgC domains, and with Skint (not present in human) possessing several TMDs [5]. Figure 1 illustrates these observations by showing the variability in the modular domain organization of the members of human BTN family, where cytoplasmic B30.2/SPRY domain is not present in BTN3A2, BTL2, BTL10, and MOG, where ERMAP and MOG do not have the IgC domains, whereas BTL2 possesses two pairs of the extracellular Ig-like domains (IgV-IgC-IgV-IgC), and MOG has two TMDs connected by a cytoplasmic linker region. Furthermore, the lengths of the inter-domain linkers found in these proteins are different.

### 2.1. Structure and Functional Disorder of the Members of Human Butyrophilin Family

#### Evaluating the Overall Disorder Status of Human Butyrophilin Family Members

First, intrinsic disorder predispositions of the members of human butyrophilin family were analyzed by a series of common disorder predictors (see Experimental Section). Results of this multi-tool disorder predisposition analysis are summarized in Table 1, which, for each human butyrophilin, shows the corresponding mean percent of predicted intrinsically disordered residues (PPIDR_mean_) values. Furthermore, for each protein, Table 1 lists the PPIDR_FIT_ values based on the PONDR^®^ FIT analysis.

Table 1 clearly shows a remarkable agreement between the PPIDR_mean_ and PPIDR_FIT_ values for individual butyrophilins. Overall, this analysis revealed that the members of human butyrophilin family are characterized by different degrees of intrinsic disorder, which range from 11.2% for BTNL2 to 21.4% for BTN1A1, as judged by their PPIDR_mean_ values. One should keep in mind though that both PONDR^®^ FIT and PPIDR_mean_ analyses represent very conservative approaches that, likely, provide the lower estimate of disorder levels in query proteins.

Typically, two arbitrary cutoffs for the levels of intrinsic disorder are used to classify proteins as highly ordered (PPIDR < 10%), moderately disordered (10% ≤ PPIDR < 30%), and highly disordered (PPIDR ≥ 30%) [21]. Therefore, data shown in Table 1 indicate that according to this classification, all human butyrophilins are predicted as moderately disordered proteins. This is an interesting, but not unexpected, observation, since, despite being transmembrane proteins (which are often rather ordered), butyrophilins are expected to contain significant levels of intrinsic disorder potentially needed for their multifunctionality.

**Figure 2:**
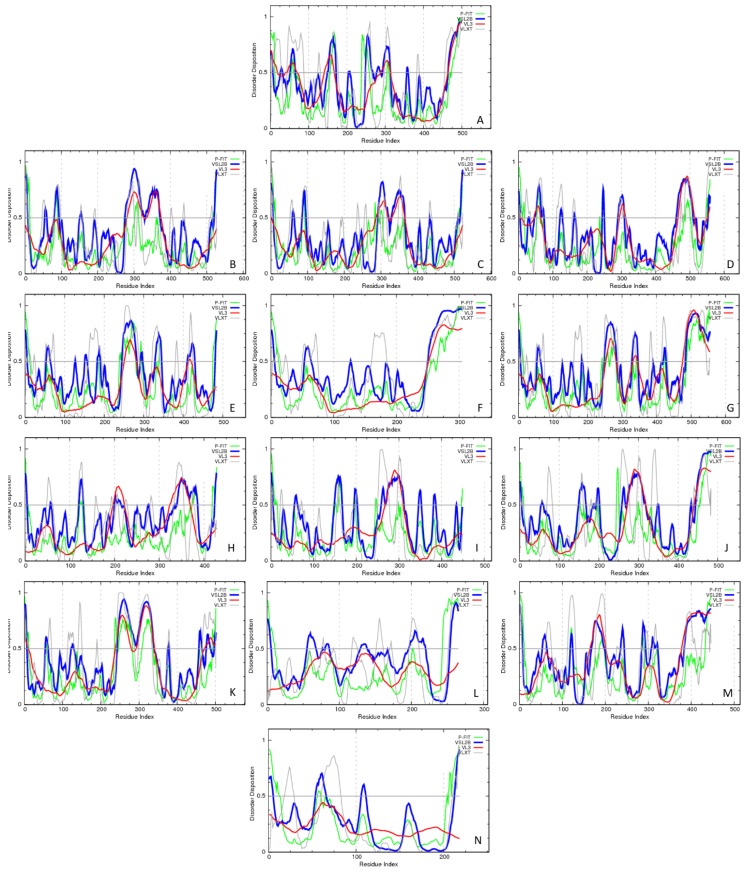
Per-residue disorder propensities of human butyrophilins as evaluated by the members of PONDR family. (**A**) BTN1A1. (**B**) BTN2A1. (**C**) BTN2A2. (**D**) BTN2A3. (**E**) BTN3A1. (**F**) BTN3A2. (**G**) BTN3A3. (**H**) BTNL2. (**I**) BTNL3. (**J**) BTNL8. (**K**) BTNL9. (**L**) BTNL10. (**M**) ERMAP. (**N**) MOG. Outputs for PONDR^®^ VLXT, PONDR^®^ VL3, PONDR^®^ VSL2, and PONDR^®^ FIT are shown by gray, red, blue, and green lines, respectively. A disorder threshold is indicated as a thin line (at score = 0.5) in all plots to show a boundary between disorder (>0.5) and order (<0.5).

Figure 2 represents the result of the multifactorial characterization of all the members of human butyrophilin family and shows their per-residue intrinsic disorder predispositions in a form of the PONDR-based disorder profiles, where disorder scores above 0.5 are considered to correspond to the disordered residues/regions. Here, disorder propensity was evaluated by four commonly used disorder predictors, PONDR^®^ VLXT [22], PONDR^®^ VL3 [23], PONDR^®^ VSL2 [24], and PONDR^®^ FIT [25]. Figure 2 clearly shows that these human proteins are characterized by highly diversified disorder profiles, and that significant parts of all human butyrophilin family members are predicted to be disordered.

Furthermore, Figure 2 and Table 1 show that each protein possesses at least one long IDPRs, positions of which varies between different members of human BTN family. Curiously, all long IDPRs are located within the cytoplasmic regions of these proteins, being preferentially found either in the middle of the proteins (typically between TMD and B30.2/SPRY domains), or at their C-terminal tails, after their B30.2/SPRY domains. This general dissimilarity of the disorder profiles is in line with the relatively low sequence conservation among the members of human butyrophilin family. In fact, Figures S1–S3 (see Supplementary Materials) represents the result of the multiple sequence alignment conducted for these 14 human proteins using the CLUSTAL Omega (1.2.4) computational platform (https://www.ebi.ac.uk/Tools/msa/clustalo/) [26,27,28]. This analysis revealed that although the intragroup sequence identities of the members of the BTN2 and BTN3 subfamilies were high (ranging from 74.03% to 84.52% and from 82.30% to 89/84%, respectively), the intergroup conservation was typically rather low, ranging from 22.37% to 74.33%, and being, on average, 36.9 ± 7.3% (see Figure S2).

These observations are in line with the known fact that the multiple sequence alignment cannot be used to identify the different butyrophilin members, and an appropriate phylogenetic analysis can be done only in the domain by domain manner [5]. Here, the identification of the orthologues and paralogues for the query sequence, its phylogenetic relations with other butyrophilin groups, and its phylogenetic group were based on the analysis of the phylogenetic tree built separately for each domain (IgV, IgC, and B30.2/SPRY) [5]. Finally, Figure S1 shows that the *N*-terminal regions of the human butyrophilin family members containing extracellular Ig-like domains are typically more conserved than their cytoplasmic domains; i.e., regions containing long IDPRs.

**Figure 3:**
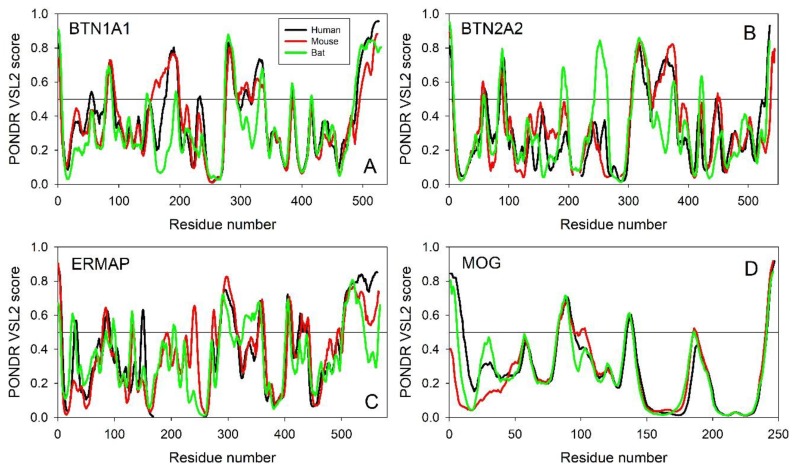
Evolutionary conservation of the peculiarities of intrinsic disorder predisposition in butyrophilins as evaluated by PONDR^®^ VSL2. (**A**) BTN1A1. (**B**) BTN2A2. (**C**) ERMAP. (**D**) MOG. Results for proteins from human, mouse, and Tasmanian devil are shown by black, red, and green lines, respectively. A disorder threshold is indicated as a thin line (at score = 0.5) in all plots to show a boundary between disorder (>0.5) and order (<0.5).

Next, to see if specific features found in the disorder profiles of the members of the human butyrophilin family are evolutionary conserved, we compared per-residue disorder propensities of some of their orthologues, using BTN1A1, BTN2A2, ERMAP, and MOG as illustrative examples, since sequences of these proteins are known for evolutionary distant species, such as human (*Homo sapiens*), mouse (*Mus musculus*), and Tasmanian devil (*Sarcophilus harrisii*). Results of this analysis are presented in Figure 3, which shows remarkable similarity of disorder profiles of these proteins in different species, indicating that the disorder predisposition peculiarities in butyrophilins are evolutionary conserved (at least since the time of marsupial divergence 160 million years ago (http://www.timetree.org/) [29]) and therefore may be related to the functionality of these proteins. Curiously, these data also indicate that disorder profiles, being well preserved among the individual butyrophilins, are highly diversified between the different members of the butyrophilin family, suggesting the role of disorder divergence in the functional diversification of these proteins.

### 2.2. Predicted Intrinsic Disorder-Based Functionality of Human Butyrophilin Family Members

#### 2.2.1. Finding Potential Disorder-Based Binding Sites, Molecular Recognition Features (MoRFs)

Among major disorder-based functions ascribed to various proteins are protein-protein interactions and molecular recognition [22,30,31,32,33,34,35,36,37,38,39,40,41,42]. Often, interactions of IDPs/IDPRs with specific partners are accompanied by binding-induced (at least partial) folding, and these structural transformations represent the core of the recognition, regulation, and signaling functions of IDPs/IDPRs [22,34,42,43,44,45,46,47,48,49].

Many IDPs/IDPRs contain disordered-embedded order-prone regions capable of undergoing the disorder-to-order transitions induced by binding to a specific partner. These disorder-based binding regions are known as Molecular Recognition Feature (MoRF), and can be computationally identified by various means, such as α-MoRF predictor that indicates the presence, within a largely disordered sequence, of a relatively short, loosely structured helical region that can undergo a disorder-to-order transition induced by binding to partners [34,47,48], or MoRFPred that accurately identifies all MoRF types (α, β, coil and complex) [50], or MoRFPred-plus computational tool that utilizes physicochemical properties and Hidden Markov Model (HMM) profiles to identify MoRFs in protein sequences [51], or ANCHOR algorithm [52,53] that relies on the pairwise energy estimation approach developed for the general disorder prediction method IUPred [54,55], or MoRF_Chibi_ algorithm that predicts MoRFs relying on the local physiochemical sequence properties [56], or DISOPRED3 that predicts IDPRs and protein-binding sites within them [57], or MFSPSSMpred (Masked, Filtered and Smoothed Position-Specific Scoring Matrix-based Predictor) [58].

Results of the application of two of these computational tools, ANCHOR and MoRF_Chibi_*Web*_, to human butyrophilins are outlined below. ANCHOR algorithm (http://anchor.enzim.hu/) generates a list of predicted disordered binding regions (which are shown in bold font in Table 1) as well as a list of filtered disorder-based binding regions, which are either short regions with length below six residues or regions with an average IUPred score below 0.1 (which are shown in the *Italic* font in Table 1) [52,53]. While looking at the human butyrophilins via ANCHOR analysis, it became obvious that all these proteins contain regions that have a significant potential to be binding sites or the ANCHOR-indicated binding sites (AIBSs, see Table 1). According to the MoRF_Chibi_*Web*_ analysis, only four human butyrophilins (BTN2A1, BTN2A2, BTN3A1, and BTNL3) are not predicted to contain MoRFs, whereas other members of this family are expected to have at least one disorder-based binding site (see Table 1). Therefore, IDPRs in butyrophilins are expected to be commonly used for protein-protein interactions.

**Figure 4:**
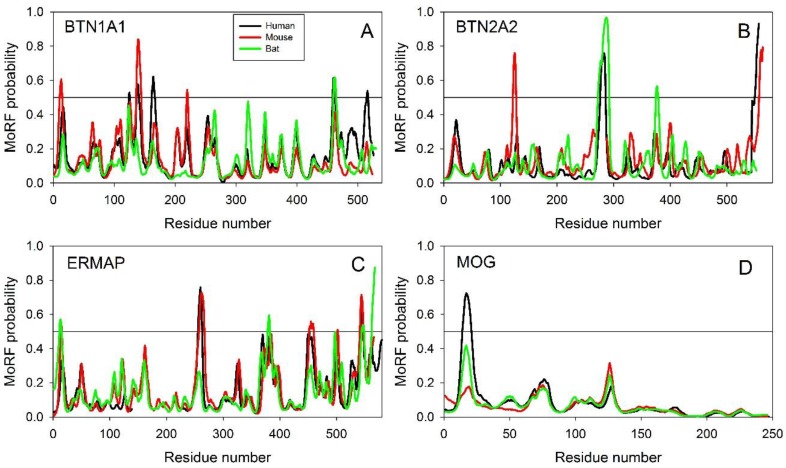
Evolutionary conservation of the disorder-based protein-protein interaction sites in butyrophilins as evaluated by ANCHOR. (**A**) BTN1A1. (**B**) BTN2A2. (**C**) ERMAP. (**D**) MOG. Results for proteins from human, mouse, and Tasmanian devil are shown by black, red, and green lines, respectively. MoRF probability was evaluated using the ANCHOR algorithm (http://anchor.enzim.hu/).

Unfortunately, the existing computational tools do not allow prediction of the disorder-based interaction regions of BTNs with some specific proteins. Therefore, we analyzed the evolutionary conservation of MoRF distribution in individual members of the butyrophilin family as a means to validate the importance of these features for the functionality of these proteins. Results of this analysis are summarized in Figure 4.

Note that to be considered as a MoRF, a region of a query protein should be longer than six residues and show MoRF probability exceeding 0.5 for its entire length [52,53]. Although analyzed proteins contain only a few regions satisfying these criteria, Figure 4 clearly shows that the individual members of the BTN family are characterized by an astounding similarity of their MoRF profiles; i.e., distributions of the MoRF probabilities in their sequences. These observations suggest that positions of not only MoRFs, but also regions with some inclination to be potential disorder-based binding sites are generally conserved over a long time (at least since the time of marsupial divergence 160 million years ago (http://www.timetree.org/) [29]).

#### 2.2.2. Predicted Intrinsic Disorder-Based Functionality of Human Butyrophilins

**Figure 5:**
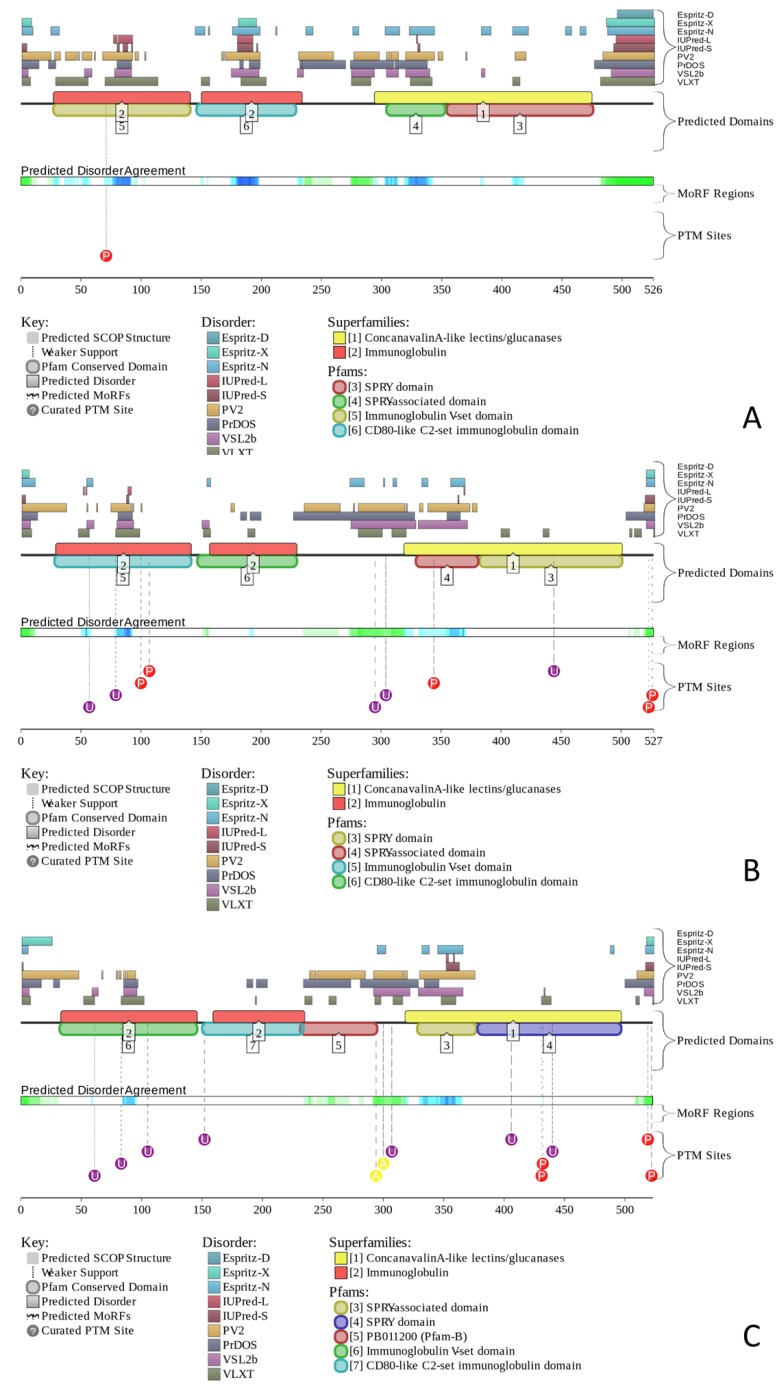
Evaluation of the functional intrinsic disorder propensity of human butyrophilins. Analysis of BT1NA1 (**A**), BT1NA2 (**B**), and BTN1A3 (**C**) was conducted by D^2^P^2^ (http://d2p2.pro/) [59]. In each plot, nine colored bars represent location of disordered regions found by different disorder predictors. A set of differently colored bars shows the location of the functional domains found by the Pfam platform, which is a database of protein families that includes their annotations and multiple sequence alignments generated using hidden Markov models [64,65,66]. Green-blue-and-white bars in the middle of the plots show the predicted disorder agreement between these nine predictors, with green and blue parts corresponding to disordered regions by consensus. Differently colored circles at the bottom of the plots show the locations of various PTMs.

Figure 5 provides further support for the abundance and functional importance of intrinsic disorder in human butyrophilins by presenting the disorder profiles generated by the D^2^P^2^ database (http://d2p2.pro/) [59]. Unfortunately, at the moment, this database contains information pertaining only to three human butyrophilins, BTN1A1, BTN1A2, and BTN1A3. However, Figure 5 clearly shows that these three human butyrophilins contain long disordered regions, which are enriched in potential disorder-based binding motifs and numerous sites of posttranslational modifications, PTMs. 

The fact that disordered regions of human BTNs contain numerous phosphorylation, acetylation, and ubiquitination sites is in agreement with the well-known notion that phosphorylation [60], as well as many other enzymatically catalyzed PTMs are preferentially located within the IDPRs [61]. These observations indicate that some disordered regions of the members of human butyrophilin family can be used as display sites [62], where the potential PTM sites are positioned to be easily accessible to the modifying enzymes. In fact, it was pointed out that the display sites, which are located within the IDPRs and function in post-translational modification, are capable of transient binding to their partner(s), modifying enzymes, that require flexibility of the substrate enabling transient but specific interaction with the active site of the modifying enzyme [62].

#### 2.2.3. Protein-Protein Interactions of Human Butyrophilins

**Figure 6:**
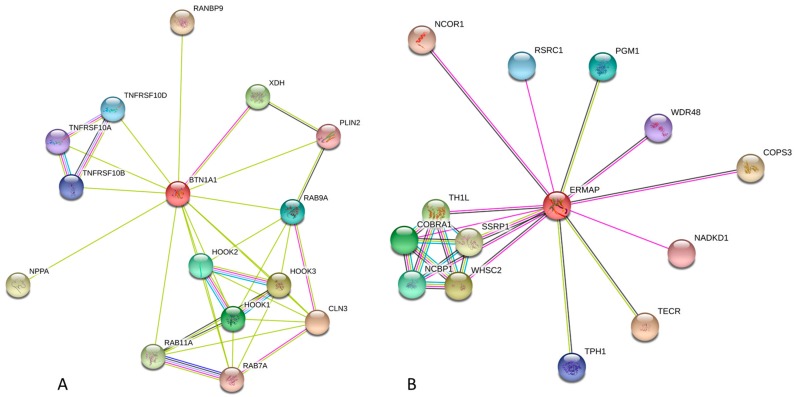
Evaluation of the interactivity of some human butyrophilins. Human BT1NA1 (**A**) and ERMAP (**B**) were analyzed by STRING platform (http://string-db.org/cgi/) [63]. An edge, which represents protein-protein interaction, may be drawn with up to 7 differently colored lines that reflect the existence of seven types of evidence used in predicting the associations. A red line indicates the presence of fusion evidence; a green line–neighborhood evidence; a blue line–co-occurrence evidence; a purple line–experimental evidence; a yellow line–text mining evidence; a light blue line–database evidence; a black line–co-expression evidence.

Results of the analysis of interactivity of the members of human butyrophilin family by STRING and BioGrid computational platforms are summarized in Table 1 and clearly show that almost all butyrophilins are expected to be highly promiscuous binders. This is further illustrated by Figure 6, where the STRING-generated protein-protein interaction networks centered at the human BTN1A1 and MOG are shown as illustration. In these networks, nodes are proteins, whereas the edges represent the predicted or known functional associations [63]. Analogous plots for other members of human butyrophilin family can be found in the Supplementary Materials (see Figure S4). High levels of predicted connectivity indicate that the members of human butyrophilin family can be considered as hub proteins, interacting with numerous partners and participating in regulation of various signaling pathways.

For example, according to the results of STRING analysis shown in Figure 6A, human BTN1A1 is engaged in interaction with ceroid-lipofuscinosis, neuronal 3 (CLN3, involved in microtubule-dependent, anterograde transport of late endosomes and lysosomes), Hook homolog 3 (HOOK3, serves as a target for the spiC protein from *Salmonella typhimurium*, acts as a component of the FTS/Hook/FHIP complex, and has several other functions), Xanthine dehydrogenase (XDH, involved in purine degradation, oxidation of hypoxanthine to xanthine, and oxidation of xanthine to uric acid); Hook homolog 1 (HOOK1, related to spermatid differentiation and cytoskeleton organization, and also serves as another component of the FTS/Hook/FHIP complex), Hook homolog 2 (HOOK2, another component of the FTS/Hook/FHIP complex and is related to the establishment and maintenance of centrosome function), Ras-related protein Rab-7A (RAB7A, a key regulator in the endo-lysosomal trafficking), Ras-related protein Rab-9A (RAB9A, a member of the RAS oncogene family involved in the transport of proteins between the endosomes and the trans Golgi network), Ras-related protein Rab-11A (RAB11A, a small GTPase regulating intracellular membrane trafficking), Tumor necrosis factor receptor superfamily member 10d (TNFRSF10D, which is a decoy with truncated death domain that is not capable of inducing apoptosis but protects against TRAIL-mediated apoptosis), Tumor necrosis factor receptor superfamily members 10b and 10a (TNFRSF10B and TNFRSF10A, respectively, serve as receptors for the cytotoxic ligand TNFSF10/TRAIL and are important for the NF- kappa-B activation and ER stress-induced apoptosis), Perilipin 2 (PLIN2, involved in development and maintenance of adipose tissue), RAN binding protein 9 (RANBP9, an adapter protein coupling membrane receptors to intracellular signaling pathways), and Natriuretic peptide A (NPPA, a hormone related to the cardiovascular homeostasis). This interactome insures involvement of BTN1A1 in a wide array of biological processes, such as lysosome organization, early endosome to late endosome transport, vacuolar transport, endosome to lysosome transport, endosomal transport, endosome organization, single-organism intracellular transport, organic substance transport, Rab protein signal transduction, cellular response to mechanical stimulus, protein processing, activation of cysteine-type endopeptidase activity involved in apoptotic process, cellular response to external stimulus, vesicle-mediated transport, activation of NF-kappaB-inducing kinase activity, protein transport, and cytoplasmic transport. In this way, BTN1A1 is controlling five biological pathways, namely apoptosis, cytokine-cytokine receptor interaction, influenza A and measles infection, and natural killer cell mediated cytotoxicity. Similarly, a list of proteins interacting with human ERMAP includes *trans*-2,3-enoyl-CoA reductase (TECR, catalyzes the last of the four reactions of the long chain fatty acids elongation cycle), Wolf-Hirschhorn syndrome candidate 2 (WHSC2, an essential component of the NELF complex related to the transcriptional pausing and negative regulation of the elongation of transcription by RNA polymerase II), TH1-like (TH1L, another essential component of the NELF complex), Cofactor of BRCA1 (COBRA1, another essential component of the NELF complex), Nuclear cap binding protein subunit 1 (NCBP1, component of the cap-binding complex), Phosphoglucomutase 1 (PGM1, participates in both the breakdown and synthesis of glucose), Arginine/serine-rich coiled-coil 1 (RSRC1 involved in both constitutive and alternative pre-mRNA splicing), Tryptophan hydroxylase 1 (TPH1, involved in step 1 of the sub-pathway that synthesizes serotonin from L-tryptophan), WD repeat domain 48 (WDR48, regulator of deubiquitinating complexes), NAD kinase domain containing 1 (NADKD1, is a mitochondrial NAD+ kinase that phosphorylates NAD+ to yield NADP+), Nuclear receptor corepressor 1 (NCOR1, regulates transcriptional repression by certain nuclear receptors), COP9 constitutive photomorphogenic homolog subunit 3 (COPS3, part of the COP9 signalosome complex involved in various cellular and developmental processes), and Structure specific recognition protein 1 (SSRP1, component of the FACT complex that acts as a general chromatin factor reorganizing nucleosomes). In this way, human ERMAP participates in multiple biological processes, such as positive regulation of viral transcription, transcription elongation from RNA polymerase II promoter, viral process, transcription from RNA polymerase II promoter, cellular biosynthetic process, and organic substance biosynthetic process. Finally, according to the STRING-based analysis, among the members of the human butyrophilin family, MOG is the most advanced regulator affecting 41 signaling pathways, such as african trypanosomiasis, allograft rejection, amoebiasis, antigen processing and presentation, asthma, autoimmune thyroid disease, cell adhesion molecules (CAMs), Chagas disease (American trypanosomiasis), cytokine-cytokine receptor interaction, cytosolic DNA-sensing pathway, Fc epsilon RI signaling pathway, graft-versus-host disease, hematopoietic cell lineage, hepatitis B, herpes simplex infection, and HTLV-I infection, inflammatory bowel disease (IBD), influenza A, intestinal immune network for IgA production, Jak-STAT signaling pathway, legionellosis, leishmaniasis, malaria, measles, natural killer cell mediated cytotoxicity, NF-kappa B signaling pathway, osteoclast differentiation, pertussis, primary immunodeficiency, rheumatoid arthritis, RIG-I-like receptor signaling pathway, *Salmonella* infection, *Staphylococcus aureus* infection, systemic lupus erythematosus, T cell receptor signaling pathway, toll-like receptor signaling pathway, toxoplasmosis, transcriptional misregulation in cancer, tuberculosis, type I diabetes mellitus, and viral myocarditis.

Therefore, butyrophilins are among the cellular multitasking proteins involved in protein-protein interaction networks (PPI), where they have multiple links and serve promiscuous binders or hubs that play crucial role in the temporal structure of the PPI networks. It is known that depending on timing of their interactivity, such highly connected proteins can be classified as “party hubs” (i.e., hubs engaged in multiple simultaneous interactions and serving as scaffolds that enable the assembly of functional modules [67]) and “date hubs” participating in multiple sequential interactions [67] and thereby connecting biological modules to each other [68]. Utilization of intrinsic disorder represents a useful mechanism for enhancing of the protein binding promiscuity [36,69,70,71,72,73]. In fact, intrinsic disorder itself and related disorder-to-order transitions define the ability of a protein to be engaged in one-to-many or many-to-one signaling, where one disordered protein/region interacts with multiple partners or multiple disordered proteins/regions bind to one partners [74]. In line with these considerations, the presence of functional IDPRs provides human butyrophilins with tactical means to serve as multifunctional hub proteins.

## 3. Discussion

Broad functionality of the members of BTN family that ranges from the crucial roles in the formation, secretion, and stabilization of milk fat globules to regulation of the immune response [5], association of these proteins with various human pathologies, such as autoimmune diseases [75,76], cardiovascular disease [77,78,79], Kawasaki disease [80], diabetes [76,81], sarcoidosis [82,83,84], multiple sclerosis [85,86], and cancer [87,88,89,90,91,92], to name a few, and their ability to protect infants against various human pathogens [93] clearly put butyrophilins to the category of moonlighting proteins. The capability of a protein to have multiple unrelated biological functions (i.e., to be a moonlighting protein) is commonly associated with the presence of intrinsically disordered protein regions (IDPRs) [37,39,49,94,95,96,97,98,99,100,101]. In fact, the presence of intrinsic disorder defines the heterogeneous spatiotemporal structure of proteins that can be described as a set of foldons, inducible foldons, semi-foldons, non-foldons, and unfoldons [49], and this heterogeneous spatiotemporal organization combined with the presence of multiple proteoforms (i.e., different proteinaceous products generated from a single gene by the allelic variations at the DNA level, such as single or multiple point mutations, indels, SNPs, or alternative splicing and other pre-translational mechanisms affecting mRNA, or a wide spectrum of posttranslational modifications (PTMs) of a polypeptide chain, or by the presence of intrinsic disorder, or as a result of functioning [102,103,104]) defines the protein multifunctionality and gives raise to the protein structure-function continuum concept [101,102].

Results of the multifactorial bioinformatics analysis of the members of human butyrophilin family conducted in this study are in line with these considerations. In fact, we are showing here that human BTNs are characterized by diversified disorder propensity and possess IDPRs that can be attributed to the multifunctionality of these important proteins. To provide some support to the findings of this study, sections below describe structural organization of the human butyrophilin family members and also disclose some evidence of the multifunctionality of human butyrophilins and discuss the therapeutic potentials of these important proteins.

### 3.1. Structural Organization of the Human Butyrophilin Family Members

Butyrophilins are known as modular transmembrane proteins containing several functional domains. For example, human BTN1A1 (UniProt ID: Q13410) has five functional regions, such as a signal peptide (residues 1–26), two immunoglobulin-like (Ig-like) domains, IgV and IgC (residues 27–138 and 148–234, respectively), a transmembrane domain (residues 243–269), and a B30.2/SPRY domain (residues 285–479). In mature protein, these functional regions constitute three topological domains, extracellular, transmembrane, and cytoplasmic (residues 27–242, 243–269, and 270–526, respectively). It was pointed out that although this domain organization was used for identification of homologous proteins, not all members of the human butyrophilin family have such domain structure. In fact, MOG does not have a B30.2 domain, whereas ERMAP does not possess the IgC domain [5]. It was also pointed out that butyrophilins are unevenly expressed in different tissues, with some of them being preferentially found in specific tissues (e.g., BTN1A1 in the secretory epithelium of the lactating mammary gland [9,10,11], BTNL2 in skeletal muscle [12], and *ERMAP* in the intestine and erythroid cells [13]), and with other butyrophilins (e.g., BTN2A1, BTN2A2, BTN3A1, BTN3A2, and BTN3A3) being abundantly present in many tissues [14,15], suggesting that the structural domains of BTN proteins may have both universal and tissue-specific functions [9]. As a result, domain by domain phylogenetic approaches were developed for the reliable identification of the different butyrophilin members [5].

Since butyrophilin genes have mosaic structure (i.e., contain introns and exons), it is not surprising that some of these proteins might have multiple isoforms generated by alternative splicing (which is a process, by which two or more mature mRNAs are produced from a single precursor pre-mRNA by the inclusion or omission of different exons [105,106]). This is the case for BTN2A2, BTN3A1, BTN3A2, BTN3A3, BTNL2, BTNL3, BTNL8, BTNL9, and MOG that are expected to have 5, 4, 3, 2, 6, 2, 7, 3, and 13 isoforms, respectively. As it was aforementioned, alternative splicing represents one of the means of the IDP/IDPR functional regulation [107]. In fact, given the ability of a single disordered protein to possess multiple functional elements, which are typically relatively short protein fragments, alternative splicing is responsible for generation of a multitude of protein isoforms with a highly diverse set of regulatory elements [107]. This phenomenon of alternative splicing-controlled functional diversification is rather common in mammals, with >60% of the mammalian genes being known to yield multiple protein isoforms [108,109,110]. Furthermore, alternative splicing is considered as one of the mechanisms enhancing protein diversity in multicellular eukaryotes for [111] by affecting various protein functions, ranging from protein-protein interactions, to ligand binding, to enzymatic activity, and posttranslational modifications [112,113,114]. Finally, time- and tissue-specific modulation of protein functions is enabled by the association of alternative splicing with protein disorder [115] that is also responsible for the rewiring of protein-protein interaction (PPI) networks, where the alternatively spliced disordered segments are used for recruiting different binding partners [116].

Despite the significant attention of researchers to butyrophilins (as of 1 January 2018 there were 2155 publications dedicated to these proteins in PubMed), the currently available structural information about human proteins is rather finite. As a matter of fact, structural characterization of human butyrophilins is limited to the presence of crystal structures of several isolated domains of some members of human butyrophilin family, whereas no structure of any full-length butyrophilin was determined as of yet. For example, for BTN3A1 (which is the best structurally characterized human butyrophilin), crystal structures are known for the Ig-like V-type 1 domain (residues 28–143 PDB ID: 4JKW), the ectodomain (residues 30–246, PDB ID: 4F80), and the intracellular B30.2/SPRY domain (residues 272–513, PDB ID: 5HM7). Also, crystal structures (PDB ID: 4F8Q and 4F8T) were solved for ectodomains of human BTN3A2 and BTN3A3 (residues 30–239 and 30–246, respectively). It is likely that the lack of the success in crystallization of the full-length butyrophilins can be, at least in part, explained by the presence of flexible (or intrinsically disordered) regions. Careful analysis of the structural information currently available for human BTN3A1 provides support to this hypothesis. In fact, although structures were solved for the isolated domains of this protein, it seems that almost entire sequence (except to the signal peptide and TMD) was structurally characterized.

However, although the whole cytoplasmic domain (residues 272–513) was used in crystallization experiments, no coordinates have been determined for the 272–322 region of this domain, indicating its high conformational dynamics (PDB ID: 5HM7). Similarly, regions 215–215 and 242–245 of the BTN3A1 ectodomain are likely to be intrinsically disordered, since they represent regions of missing electron density and no coordinates have been determined for them (PDB ID: 4F80) [117].

**Figure 7:**
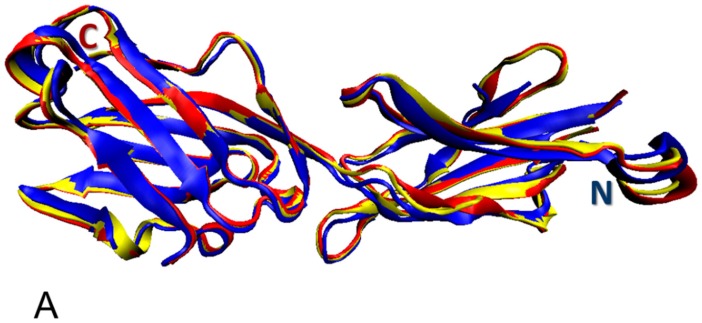

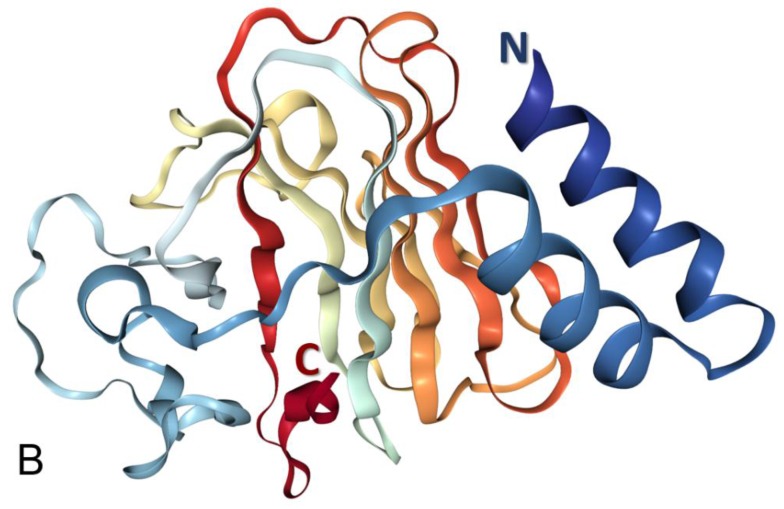
(**A**) Multiple structural alignments of the crystal structures of ectodomains of human BTN3A1 (residues 30–246, PDB ID: 4F80; yellow structure), BTN3A2 (residues 30–239, PDB ID: 4F8Q; blue structure), and BTN3A3 (residues 30–246, PDB ID: 4F8T; red structure). Structures were aligned using the MultiProt tool [118]. (**B**) Crystal structure of the intracellular B30.2/SPRY domain of human BTN3A1 (residues 272–513, PDB ID: 5HM7).

Figure 7 provides an outlook of the currently available structural information pertaining to the members of human butyrophilin family. It indicates a very close structural similarity of the ectodomains in BTN3 subfamily and shows that the intracellular B30.2/SPRY domain of BTN3A1 contains high proportion of irregular structure that accounts for 60% of its sequence, suggesting the presence of high level of structural flexibility in this domain.

These observations are in agreement with an earlier structural and functional comparison of the extracellular domains of the three BTN3A isoforms that clearly showed that structurally, these recombinant domains (each containing two Ig-like domains: an *N*-terminal Ig-V like domain and a C-terminal Ig-C type domain) were highly similar, differing from each other by the backbone C_α_ RMSD of no more than 0.9 Å^2^ [117]. This study also revealed that the BTN3A ectodomains exist preferentially in a form of a V-shaped homodimer, where the protomers are associated by a symmetrical interface at the membrane proximal region of their C domains [117].

Furthermore, multiple studies suggested that conformational flexibility of the intracellular domain could be very important for the functionality of human BTN3A1, which is known to interact specifically with various small phosphorus-containing molecules, such as prenyl-pyrophosphate (IPP) [119,120], (E)-4-hydroxy-3-methyl-but-2-enyl pyrophosphate (HMBPP) [121], or the phosphoantigen prodrugs [122,123], and initiates transmembrane signaling and activates BTN-responsive cells, thereby defining sensitivity of the Vγ9Vδ2 T cells to phosphoantigens of bacterial, viral, or malignant origin [119,124,125,126,127]. Mature BTN3A1, which is generated by the removal of its signal peptide, is a ∼54 kDa, type I receptor glycoprotein possessing an extracellular IgV/IgC domain, a transmembrane domain (TMD), and an intracellular region containing a B30.2 (PRY/SPRY) domain attached to the TMD by a 68-residue-long juxtamembrane (JM) region.

Combined NMR and SAXS analysis revealed that binding of HMBPP to the full-length intracellular domain not only affect the B30.2 (PRY/SPRY) domain, but also introduce perturbation of residues in the JM region [123,128]. In fact, these studies showed that in the unbound state, the JM region was predominantly random and flexible, but gained some helical structure after the HMBPP binding to the B30.2 (PRY/SPRY) domain leading to the formation of a clamped B30.2/HMBPP/JM complex [128].

### 3.2. Butyrophilins and Immune Response

BTN, being one of the milk protein responsible for the fat transport, is considered structurally as a type I membrane protein from the immunoglobulin V domain superfamily. In addition, the extracellular domains of BTNs share structural similarity with the family of the B7 costimulatory molecules that includes numerous signaling molecules playing principal roles in the T-cell regulation, activation, and tolerance. This family includes receptors and ligands implicated in T-cell co-stimulation, such as B7.1 (CD80) and B7.2 (CD86) and their receptors, as well as immune-checkpoint molecules of T-cell, such as PD-L1 (CD274) and PD-L2 (CD273) and their receptors, such as PD-1 (CD279) [5,117,129]. The phylogenetic analysis of the BTNs revealed that both BTN and B7 family have a common ancestor, suggesting that BTN could have an immunological functions [130]. In agreement with this hypothesis, BTN1A1 and BTN2A2 were shown to inhibit the anti-CD3-induced IL-2 production by human T-lymphocytes [7]. Also, human T-cell proliferation can be inhibited by BTN3A1 in a dose-dependent manner, and this BTN was shown to bind to the activated human T-cells [119,129].

BTN3A1 (CD277) is acting as a strong antigen-presenting molecule of both, IPP (prenyl-pyrophosphate, which is a product of eukaryotic cells) [119,120] and HMBPP ((E)-4-hydroxy-3-methyl-but-2-enyl pyrophosphate, which is a product of cancer cells and certain selected pathogenic bacteria, such as *Mycobacterium bovis*, *Mycobacterium tuberculosis*, *Listeria monocytogenes*, as well as certain species of malaria protozoa) for the γδ T-cells [121]. The crystal structures of BTN3A1 complexed with IPP or HMBPP were solved showing that both phosphorylated antigens are specifically bound in a shallow groove, revealing the similarity of the immunoglobulin V domain in the bound states, and suggesting the formation of a platform, where both the antigen and BTN3A1 might interact with the T-cell receptor (TCR) [127]. Curiously, binding of both phosphorylated antigens to BTN3A1 was accompanied by the noticeable structural changes in the BTN3A1 immunoglobulin V domain [127]. It was also shown that the Vγ9Vδ2 (and Vγ2Vδ2 as well) T-cells can recognize the HMBPP and/or IPP molecules in the context of BTN3A1, which may facilitates binding of the two proteins, independently of the CD1d molecules [131]. Therefore, the principles of the binding may be similar in γδ T-cells as well as in αß T-cells, where after fixing the antigen in a groove by the non-covalent interactions, both antigen and antigen-presenting molecules are recognized by the TCR domain [6,92,127]. However, others researchers did not see this similarity [131]. Furthermore, BTN3 molecules can also contribute to the maintenance of the immune system through an inhibition of the excessive cellular immune responses [132].

The Vγ9Vδ2 (and Vγ2Vδ2 as well) T-cells are a subset of the human γδ T-lymphocytes expressing TCR γ9 that are HLA-unrestricted and CD1-independent. They are able to recognize both protein and the non-peptide molecules (BTN3A1/CD277 and phosphoantigens (PAgs), respectively). These T-cells constitute about 65–90% of the γδ T-lymphocytes and 1–10% of the total blood T-lymphocytes. They circulate in peripheral blood of human and non-human primates only [133]. However, circulation could be expanded in human infected patients with pathogens, such as *Plasmodium falciparum* [134,135,136,137,138] or *M. tuberculosis* [139,140], and also in the patients with lymphoid malignancies [141,142]. These T-cells may play a dual role in patients with malarial infection, since they can promote the pathological events and contribute, together with the BTN molecules, to the control of the parasites density [133,143]. These various responses against wide range of different structures raised an important functional question, namely: are these different structures induce the same intracellular activation signaling cascade? Hence the highly specific response of TCR Vγ9Vδ2^+^ T-cells to PAgs was revealed to be dependent not only on the expression of the BTN3A molecules by antigen-presenting cells, but also on the presence of a product(s) of the *Chr6* gene required for sufficient PAg-mediated activation of Vγ9Vδ2 T lymphocytes [127,144,145]. The answer to this important question was retrieved through a simple experimental design, which suggested that TCR γ9+ T-lymphocytes are able to recognize both PAgs and BTN3A1/CD277, leading to the activation of the same intracellular signaling pathways that included ZAP70, PLCγ2, Akt, NFĸB p65, MAPK p38, and Erk1 [144].

Many reports have established that BTN molecules play a set of crucial roles in controlling the biological activity of T-lymphocytes via regulating their proliferation and activation through anti-CD3 [146,147]. Furthermore, human immature dendritic cells (iDCs) can be modulated by the BTN2A1 molecule via binding to the intercellular adhesion molecule-3-grabbing non-integrin of iDCs (DC-SIFN) [148]. In addition to the aforementioned immune-regulatory functions, BTNs also work as co-inhibitory and co-stimulatory (agonist/antagonistic) molecules in different complexes as recently reviewed [131]. These functions of BTNs indicate that these proteins may serve as candidate therapeutic targets in several diseases, such as cancer, transplant tolerance, and inflammatory diseases. Similar to some members of the B7 family, members of the BTN family can also interact with unknown binding partners in different cell types inducing stimulatory or inhibitory responses [131]. Recently, BTN2A2 encoding genes in both human and mouse have been found to be regulated by the same regulatory factors (class II trans-activator and regulatory factor X) of major histocompatibility complex class II transcription factors [149], which suggested a role of BTN2A2 in the T-cell immunity, likely as co-inhibitor molecules during the T-cell-mediated immunity.

### 3.3. Therapeutic Potentials of Butyrophilins

Purified BTN was shown to interact with the F4ac molecules of the enterotoxigenic *E.coli* (ETEC) strains and prevented attachment of this bacteria to the neonatal porcine intestinal enterocytes in vitro, suggesting that utilization of this protein may reduce the diarrhea in piglets [150,151]. As aforementioned, the stimulation of Vγ9Vδ2^+^ T-cells by IPP or HMBPP antigens was dependent on the BTN. It is known that both antigens are secreted by certain microbial pathogens. Therefore, the butyrophilin/ Vγ9Vδ2^+^ T-cells may play a crucial role not only in the defense against *M. tuberculosis*, *M. bovis*, and *L. monocytogenes* during primary infection, but also, they mount the recall expansion in systemic infection or in lung compartments after reinfection with *M. tuberculosis* or other related pathogens [133]. This adaptive-like immune response (recall-like expansion) is characterized by a memory-like pattern for a long period, high magnitude, and faster secondary immune response than primary immune response [133]. This recall-like expansion is characterized by specific cytokine profile.

The autoantibodies raised against nine different neuron-specific antigens were detected in sera of autistic children. Those autoantibodies were able to significantly cross-react with the BTN peptide 89–109 and some other neurological antigens [152]. Is this an indication that BTN may play a role in autoimmune response modulation? An elegant study [153] has documented the presence of a cross-reactivity or molecular mimicry between myelin the oligodendrocyte glycoprotein (MOG) and the extracellular domain of BTN. In this study, the antibodies raised against MOG were used in a panel of assays analyzing the experimental autoimmune encephalomyelitis via T-cell dependent immune response, as well as in the MHC-restricted immune response produced against BTN. The immune responses products against both antigens (MOG and BTN) were mutually cross-reactive. This study also demonstrated that the intranasal and intravenous treatment with BTN peptide can conversely abrogate MOG-induced autoimmune encephalitis [153]. The molecular mimicry between MOG and butyrophilin let Mana et al. to extend those observations and demonstrate that the treatment of C57BL/6 mice with BTN either after or before MOG immunization caused a prevention and suppression of the clinical manifestation of experimental autoimmune encephalomyelitis (EAE) [154]. This BNT-dependent prevention/suppression was shown to depend on a significant reduction in the production of both proliferation and Th1-related cytokines (IFN-γ, IL-2, IL-12, GM-CSF) in response to MOG. This significant prevention was consistently associated with the up-regulation of the IL-10 production too. Another interesting point was found when the adoptive of BTN-specific T-lymphocytes were transferred before the active-immunization with MOG, which resulted in a transitory reduction of the EAE clinical symptoms [154]. These results may reflect the presence of a link between the clinical improvement caused by the treatment with BTN and the presence of both the anergy (i.e., immune unresponsiveness) and the regulatory cells that secrete high concentration of IL-10 [154]. This may suggest that the consumption of milk and milk products, especially the dietary BTN, could modulate the pathogenic autoimmune response to MOG in multiple sclerosis [153].

The binding of PAgs to the extra- or intracellular domains of the cell surface membrane molecule BTN3A1 may lead to a novel structure or conformation that seems to be needed and subsequently detected by the T-lymphocytes expressing the Vγ9Vδ2 TCR, thereby triggering their cytokine production and/or cytotoxicity [155,156]. This induction scenario has been analyzed both in vitro and in vivo and against a broad range of malignant cells including glioblastoma multiforme (GBM) [155,156,157]. Ligands of the NKG2D stimulatory receptor are not expressed by the most normal tissues but are abundantly present in many tumor-cell types, including GBM. NKG2D is a C-type lectin receptor expressed on NK, γδ T-lymphocytes, and CD8+ αβ T-lymphocytes. Many reports demonstrated a distinctive contribution of NKG2D to the γδ T-cell-mediated tumor immune surveillances in both human and mice [158,159]. For example, mice lacking γδ T-cells are highly susceptible to the cutaneous carcinogenesis, and this susceptibility seems to be regulated by the NKG2D expressed on the γδ T-cells [160]. Furthermore, NKG2D ligands seem to represent key molecular determinants of the immune recognition of oncogenic stress [161]. Recent study of Chitadze et al. revealed that NKF2DLs expression on the cells was increased, as well as the GBM cells were sensitized to γδ T-cell-mediated lysis after treatment with temazolomide (TMZ) [157]. In addition, stimulation of the γδ T-cells with the pyrophosphate antigens strongly enhanced their cytotoxic activity against GBM through the TCR-dependent profile [157]. Furthermore, Benyamine et al. demonstrated that the use of BTN3A1 can improve the Vγ9Vδ2 T-cells-based immunotherapy of acute myeloid leukemia [162].

Two experimental models were designed by Werter et al. to eliminate cancer cells via Vγ9Vδ2 T-cell/BTN3A1, which is working as an antigen presenting cell for the invariant natural killer T-cells (iNKT) [163]. Both approaches were shown to result in the Vγ9Vδ2 T-cells that have the capacity to act as antigen presenting cells for iNKT via trogocytosis of membrane-associated antigens, which can act as APS to propagate Th1 biased anti-tumor immune responses within tumor microenvironment [163]. These models are in agreement with the previous studies, which demonstrated that the PAg-induced Vγ9Vδ2 T-cells could recognize and kill a variety of cancer cells both in vitro as well as in vivo, such as nasopharyngeal, colon, pancreatic adenocarcinomas, breast carcinoma, melanoma, as well as the large number of hematological tumors [155,164,165,166,167].

Although a few studies have explored the association between human BTN3 molecules and ovarian cancer [89,92,168], recently Lebrero-Fernández et al. has been able to clarify the connection between BTN/BTNL molecules and control of the T-lymphocyte-based immune responses, which are genetically associated with the inflammatory disorders and cancer [87]. This connection was established through the analysis of gene expression levels of both BTN and BTNL in human and mouse intestinal tissues [87]. These valuable results may help in development approaches for predicting of the intestinal inflammation and/or cancer.

In a series of reports from the research group of Prof. Yoshiji Yamada at Inabe General Hospital (Japan) a link has been found between the polymorphisms of the BTN2A1 gene and several medical symptoms and deficiencies [81,169,170,171,172,173,174,175,176,177,178,179]. In fact, in these studies, the C-T polymorphism of the *BTN2A1* gene allele was significantly associated with the myocardial infarction [169,170], dislipidema [171,172,173], chronic kidney diseases (CKD) [172,174,175,176], hypertension [177,178,179], type 2 diabetes mellitus [81], and metabolic syndrome [180] in Japanese individuals and in East Asian populations.

## 4. Experimental Section

### 4.1. Dataset

In this article, we analyzed fourteen members of the human butyrophilin family, BTN1A1 (UniProt ID: Q13410), BTN2A1 (UniProt ID: Q7KYR7), BTN2A2 (UniProt ID: Q8WVV5), BTN2A3 (UniProt ID: Q96KV6), BTN3A1 (UniProt ID: O00481), BTN3A2 (UniProt ID: P78410), BTN3A3 (UniProt ID: O00478), BTNL2 (UniProt ID: Q9UIR0), BTNL3 (UniProt ID: Q6UXE8), BTNL8 (UniProt ID: Q6UX41), BTNL9 (UniProt ID: Q6UXG8), BTNL10 (UniProt ID: A8MVZ5), ERMAP (UniProt ID: Q96PL5), and MOG (UniProt ID: Q16653). Sequences of these proteins in FASTA format were extracted from the UniProt database [181]. We also analyzed evolutionary conservation of some of the members of the butyrophilin family. To this end, we compared different sequence-based features of human BTN1A1 (UniProt ID: Q13410), BTN2A2 (UniProt ID: Q8WVV5), ERMAP (UniProt ID: Q96PL5), and MOG (UniProt ID: Q16653) with the corresponding features of their orthologues from mouse (*Mus musculus*), and Tasmanian devil (*Sarcophilus harrisii*). The analyzed mouse proteins were Btn1a1 (UniProt ID: Q62556), Btn2a2 (UniProt ID: A4QPC6), Ermap (UniProt ID: Q9JLN5), and Mog (UniProt ID: Q61885). The analyzed Tasmanian devil proteins were Btn1a1 (UniProt ID: G3VLA4), Btn2a2 (GI ID: XP_012404130.1), Ermap (GI ID: XP_023356020.1), and Mog (GI ID: XP_012404306).

### 4.2. Methods

#### 4.2.1. Multiple Sequence Alignment

Amino acid sequence of the members of human butyrophilin family were aligned using the CLUSTAL Omega (1.2.4) computational platform for multiple sequence alignments (https://www.ebi.ac.uk/Tools/msa/clustalo/) [26,27,28], utilizing the default settings.

#### 4.2.2. Evaluation of Intrinsic Disorder Predisposition of Human Butyrophilins

In this study, we utilized a multi-tool disorder predisposition analysis and used ESpritz-NMR and ESpritz-XRay [182], IUPred-Long and IUPred_short [54], DisEMBL-465 and DisEMBL-HL [183], GlobPlot [184], and PONDR^®^ VSL2 [23,185]), access to which is provided by the MobiDB database (http://mobidb.bio.unipd.it/) [186,187]. For each human butyrophilin, the corresponding results of this multi-tool disorder predisposition analysis were converted to mean PPIDR percent of predicted intrinsically disordered residues (PPIDR_mean_). The corresponding PPIDR_mean_ values were calculated by averaging outputs of the DisEMBL-465, DisEMBL-HL, ESpritz-NMR, ESpritz-XRay, GlobPlot, IUPred-long, IUPred-short and PONDR^®^ VSL2.

For each protein, we also used PONDR^®^ FIT analysis [25]. PONDR^®^ FIT is a metapredictor that combines six individual predictors, which are PONDR^®^ VLXT [188], PONDR^®^ VSL2 [24], PONDR^®^ VL3 [23], FoldIndex [189], IUPred [54], TopIDP [190]. It was shown that PONDR^®^ FIT is moderately more accurate than each of the component predictors [25].

We further characterized the per-residue intrinsic disorder predispositions butyrophilins in a form of the PONDR-based disorder profiles. To this end, each human butyrophilin was analyzed by PONDR^®^ VLXT [22], PONDR^®^ VL3 [23], PONDR^®^ VSL2 [24], and PONDR^®^ FIT [25]. These tools were selected based on the peculiarities of their sensitivities and performance. Although PONDR^®^ VLXT is not among the most accurate predictors, this tool has a high sensitivity to local sequence peculiarities, which are often associated with disorder-based interaction sites [22]. According to the comprehensive assessment of *in silico* predictors of intrinsic disorder [191,192], PONDR^®^ VSL2 [24] is one of the more accurate stand-alone disorder predictors. PONDR^®^ VL3 is known to perform well for finding long IDPRs [23], and advantages of the PONDR^®^ FIT metapredictor [25] were already mentioned.

#### 4.2.3. Evaluation of the Functionality of Intrinsically Disordered Regions in Human Butyrophilins

To find disordered regions of human butyrophilins that have a potential to fold at interaction with binding partners and therefore can serve as disorder-based binding sites known as molecular recognition features (MoRFs), MoRF_Chibi_ algorithm was used that predicts MoRFs relying on the local physiochemical sequence properties [56].

Potential binding sites in disordered regions of human butyrophilins were also identified by the ANCHOR algorithm [52,53]. This approach relies on the pairwise energy estimation approach developed for the general disorder prediction method IUPred [54,55], being based on the hypothesis that long regions of disorder contain localized potential binding sites that cannot form enough favorable intrachain interactions to fold on their own, but are likely to gain stabilizing energy by interacting with a globular protein partner [52,53]. Regions of a protein suggested by the ANCHOR algorithm to have significant potential to be binding sites are the ANCHOR-indicated binding sites (AIBSs).

We also utilized the D^2^P^2^ database (http://d2p2.pro/), which is a community resource of pre-computed disorder predictions for proteins from completely sequenced genomes [59]. The advantage of D^2^P^2^ database is in its use of the outputs of several disorder predictors (PONDR^®^ VLXT [188], IUPred [54], PONDR^®^ VSL2B [23,185], PrDOS [193], ESpritz [182], and PV2 [59]) combined with the information on the curated sites of various posttranslational modifications and on the location of predicted disorder-based potential binding sites. This combination makes presented results for a query protein rather intuitive.

The interactivity of human butyrophilins was further evaluated by STRING (Search Tool for the Retrieval of Interacting Genes), which is the online database resource, that provides both experimental and predicted interaction information [63]. STRING produces the network of predicted associations for a particular group of proteins. The network nodes are proteins, whereas the edges represent the predicted or known functional associations, where seven types of evidence are used in predicting the functional associations [63]. In addition to STRING, we also used The Biological General Repository for Interaction Datasets (BioGrid), which is a public database containing genetic and protein interaction data from model organisms and humans (http://www.thebiogrid.org) [194].

## 5. Conclusions

Despite the fact that human butyrophilins have a multitude of important biological functions and are engaged in the pathogenesis of several diseases, structural information about these modular transmembrane proteins is rather limited. Furthermore, no systematic in silico analysis was ever conducted to evaluate the intrinsic disorder propensity of human butyrophilins. The goal of this article was to show how intrinsic disorder is encoded in the sequences of fourteen members of the human butyrophilin family, and to look at the peculiarities of functional intrinsic disorder in these proteins. Our analyses revealed that although all members of human butyrophilin family contain noticeable levels of intrinsic disorder, they are characterized by highly diversified disorder propensities and possess very different disorder profiles. We show also that although disorder predisposition is highly diverged between the family members, disorder profiles of these proteins in different species are characterized by the remarkable similarity, indicating that the disorder predisposition peculiarities in individual butyrophilins are evolutionary conserved at least since the time of marsupial divergence 160 million years ago and therefore may be related to the functionality of these proteins. Furthermore, since disorder profiles are well preserved among the individual butyrophilins, but are highly diversified in the different members of the butyrophilin family, one can assume that such diversification of the intrinsic disorder propensity represents a molecular means for the functional diversification of these proteins. In fact, our study revealed that IDPRs in butyrophilins can be used for interaction of these proteins with various binding partners and serve as primary targets for various PTMs. Curiously, we found that the individual members of the BTN family are characterized by a remarkable similarity of their MoRF profiles, which represent the sequence distributions of the probability for being engaged in the disorder-based interactions. Therefore, findings of this study clearly indicate that intrinsic disorder plays important roles in function and evolution of the butyrophilin family and therefore should be taken into account in structural and functional studies of these proteins.

## Figures and Tables

**Table 1:** Evaluation of intrinsic disorder propensity of the members of human butyrophilin family.

Name/UniProt ID (Protein Length) ^a^	PPIDR_mean_^b^	PPIDR_FIT_^c^	Long IDPRs ^d^	AIBS ^e^	MoRF_Chibi_*Web*_^f^	Number of Binding Partners ^g^
BTN1A1/Q13410 (500 a.a.r.)	21.4 ± 4.1	17.6	44–87 456–500	*124–125 137–140 162–166 459–462 515–517*	481–493	14 (3)
BTN2A1/Q7KYR7 (499 a.a.r.)	12.3 ± 4.8	9.0	303–343	*22–23 260–267*	N.P.	3 (38)
BTN2A2/Q8WVV5 (491 a.a.r.)	10.2 ± 3.3	8.1	255–333	*277–285*	N.P.	8 (34)
BTN2A3/Q96KV6 (559 a.a.r.)	13.3 ± 2.9	11.3	461–509	*18–23 123–124 136–139 551–562*	523–558	Unknown
BTN3A1/O00481 (484 a.a.r.)	14.5 ± 3.8	16.5	248–290	*125–128 337–338 380–391*	N.P.	12 (14)
BTN3A2/P78410 (305 a.a.r.)	13.5 ± 2.5	18.0	150–305	*125–128*	278–304	6 (Unknown)
BTN3A3/O00478 (555 a.a.r.)	19.7 ± 2.1	20.4	247–289 481–545	**495–513** **549–563 575–584** *125–128 381–388 464–467 537–540*	524–531 545–555	4 (9)
BTNL2/Q9UIR0 (428 a.a.r.)	11.2 ± 2.4	7.5	324–369	354–361 *265–269 341–244*	36–41 359–362	14 (Unknown)
BTNL3/Q6UXE8 (449 a.a.r.)	11.5 ± 3.3	8.0	268–310	*254–258 353–355*	N.P.	49 (10)
BTNL8/Q6UX41 (483 a.a.r.)	18.8 ± 3.5	10.6	272–318 441–483	*241–258*	454–464 473–482	26 (42)
BTNL9/Q6UXG8 (501 a.a.r.)	19.8 ± 2.5	20.0	243–339	**324–332** *533–535*	485–501	38 (Unknown)
BTNL10/A8MVZ5 (265 a.a.r.)	12.1 ± 3.9	12.8	197–219	**126–131** *265–273*	1–6 40–44	Unknown
ERMAP/Q96PL5 (446 a.a.r.)	17.6 ± 4.1	11.0	378–446	*168–175 364–365 455–459*	34–38 422–429	13 (14)
MOG/Q16653 (218 a.a.r.)	12.1 ± 3.3	13.8	61–82	*14–20*	1–9 19–25 35–56 64–72	85 (1)

^a^ Length (in amino acid residues) of the mature protein after the removal of the signal peptide; ^b^ PPIDR_mean_ are the mean disorder PPIDR values calculated for each protein by averaging their DisEMBL-465, DisEMBL-HL, ESpritz-NMR, ESpritz-XRay, GlobPlot, IUPred-long, IUPred-short and PONDR^®^ VSL2b PPIDR values from the corresponding MobiDB pages; ^c^ PPIDR_FIT_ are the PPIDR values from PONDR^®^ FIT analysis; ^d^ Positions of long IDPRs from the PONDR page are indicated; ^e^ Positions of AIBSs (which are ANCHOR-identified binding sites) are indicated ^f^ Positions intrinsic disorder-based binding sites, molecular recognition features (MoRFs), identified by MoRF_Chibi_*Web*_ are indicated; ^g^ Number of binding partners was evaluated by STRING and BioGrid, with BioGrid data shown in brackets.

## Supplementary Materials

The following are available online, Figure S1: Multiple sequence alignment of human butyrophilin family members by the CLUSTAL Omega (1.2.4) algorithm, Figure S2: Percent Identity Matrix created by Clustal 2.1 for human butyrophilin family members, Figure S3: Phylogenetic tree created for the human butyrophilin family members by the CLUSTAL Omega (1.2.4) algorithm, Figure S4: Analysis of the interactivity of the human BTN2A1 (A), BTN2A2 (B), BTN3A1 (C), BTN3A2 (D), BTN3A2 (E), BTNL2 (F), BTNL8 (G), BTNL9 (H), and MOG (I) by STRING platform (http://string-db.org/cgi/).
