# Supplementary material for: Prediction of Disordered Regions and Their Roles in the Anti-Pathogenic and Immunomodulatory Functions of Butyrophilins
Source: Molecules. 2018 Feb 4;23(2):328. doi: 10.3390/molecules23020328 (PMC6017450; doi:10.3390/molecules23020328)
Supplement: Supplementary file 1 [file molecules-23-00328-s001.pdf]

Supplementary Materials

Prediction of disordered region and their roles in the anti-pathogenic and immunomodulatory functions of butyrophilins

Elrashdy M. Redwan[1,2,*], Ahmed M. Al-Hejin[1], Hussein A. Almehdar[1], Abdelrahman M. Elsaway[2] and Vladimir N. Uversky[1,4,5,*]

[1] *Department of Biological Science, Faculty of Science, King Abdulaziz University, Jeddah, PO Box 80203, Jeddah 21589, Saudi Arabia; E-Mail: lradwan@kau.edu.sa*

[2] *Therapeutic and Protective Proteins Laboratory, Protein Research Department, Genetic Engineering and Biotechnology Research Institute GEBRI, City for Scientific Research and Technology Applications, New Borg EL Arab 21934, Alexandria, Egypt.*

[3] *Microbiology Department, Faculty of Medicine, Al-Azhar University, Cairo, Egypt.*

[4] *Department of Molecular Medicine and USF Health Byrd Alzheimer's Research Institute, Morsani College of Medicine, University of South Florida, Tampa, Florida 33612, USA; E-Mail: vuversky@health.usf.edu*

[5] *Laboratory of New Methods in Biology, Institute for Biological Instrumentation, Russian Academy of Sciences, 142290 Pushchino, Moscow Region, Russia*

***** Authors to whom correspondence should be addressed; E-Mails: vuversky@health.usf.edu (V.N.U.) and lradwan@kau.edu.sa (E.M.R.)

Figure S1. Multiple sequence alignment of human butyrophilin family members by the CLUSTAL Omega (1.2.4) algorithm

```
sp|Q13410|BT1A1_HUMAN      ----APFDVIGPPEPILAVVGEDAELPCRLSPNASAEHLELRWFRKKV---SPAVLVHRD      53
sp|Q7KYR7|BT2A1_HUMAN      -----QFIVVGPTDPILATVGENTTLRCHLSPEKNAEDMEVRWFRSQF---SPAVFVYKG      52
sp|Q8WVV5|BT2A2_HUMAN      -----QFTVVGPANPILAMVGENTTLRCHLSPEKNAEDMEVRWFRSQF---SPAVFVYKG      52
sp|Q96KV6|BT2A3_HUMAN      -----QVTVVGPTDPILAMVGENTTLRCCLSPEENAEDMEVRWFQSQF---SPAVFVYKG      52
sp|O00481|BT3A1_HUMAN      -----QFSVLGPSGPILAMVGEDADLPCHLFPTMSAETMELKWVSSSL---RQVVNVYAD      52
sp|P78410|BT3A2_HUMAN      -----QFSVLGPSGPILAMVGEDADLPCHLFPTMSAETMELKWVSSSL---RQVVNVYAD      52
sp|O00478|BT3A3_HUMAN      -----QFSVLGPSGPILAMVGEDADLPCHLFPTMSAETMELRWVSSSL---RQVVNVYAD      52
sp|Q9UIR0|BTNL2_HUMAN      -----DFRVIGPAHPILAGVGEDALLTCQLLPKRTTMHVEVRWYRSEP---STPVFVHRD      52
sp|Q6UXE8|BTNL3_HUMAN      -----QWQVTGPGKFVQALVGEDAVFSCSLFPETSAEAMEVRFFRNQF---HAVVHLYRD      52
sp|Q6UX41|BTNL8_HUMAN      -----QWQVFGPDKPVQALVGEDAAFSCFLSPKTNAEAMEVRFFRGQF---SSVVHLYRD      52
sp|Q6UXG8|BTNL9_HUMAN      ---SSEVKVLGPEYPILALVGEEVEFPCHLWPQLDAQQMEIRWFRSQT---FNVVHLYQE      54
sp|A8MVZ5|BTNLA_HUMAN      SIWKADFDVTGPHAPILAMAGGHVELQCQLFPNISAEDMELRWYRCQP---SLAVHMHER      57
sp|Q96PL5|ERMAP_HUMAN      -------HAGDAGKFHVALLGGTAELLCPLSLWPGTVPKEVRWLRSPFPQRSQAVHIFRD      53
sp|Q16653|MOG_HUMAN        ----GQFRVIGPRHPIRALVGDEVELPCRISPGKNATGMEVGWYRPPF---SRVVHLYRN      53
                                   . .      *  *  . : * :     :   *: :           * :.

sp|Q13410|BT1A1_HUMAN      GREQEAEQMPEYRGRATLVQDGIAKGRVALRIRGVRVSDDGEYTCFFREDGSYEEALVHL      113
sp|Q7KYR7|BT2A1_HUMAN      GRERTEEQMEEYRGRTTFVSKDISRGSVALVIHNITAQENGTYRCYFQEGRSYDEAILHL      112
sp|Q8WVV5|BT2A2_HUMAN      GRERTEEQMEEYRGRITFVSKDINRGSVALVIHNVTAQENGIYRCYFQEGRSYDEAILRL      112
sp|Q96KV6|BT2A3_HUMAN      GRERTEEQKEEYRGRTTFVSKD-SRGSVALIIHNVTAEDNGIYQCYFQEGRSCNEAILHL      111
sp|O00481|BT3A1_HUMAN      GKEVEDRQSAPYRGRTSILRDGITAGKAALRIHNVTASDSGKYLCYFQDGDFYEKALVEL      112
sp|P78410|BT3A2_HUMAN      GKEVEDRQSAPYRGRTSILRDGITAGKAALRIHNVTASDSGKYLCYFQDGDFYEKALVEL      112
sp|O00478|BT3A3_HUMAN      GKEVEDRQSAPYRGRTSILRDGITAGKAALRIHNVTASDSGKYLCYFQDGDFYEKALVEL      112
sp|Q9UIR0|BTNL2_HUMAN      GVEVTEMQMEEYRGWVEWIENGIAKGNVALKIHNIQPSDNGQYWCHFQDGNYCGETSLLL      112
sp|Q6UXE8|BTNL3_HUMAN      GEDWESKQMPQYRGRTEFVKDSIAGGRVSLRLKNITPSDIGLYGCWFSSQIYDEEATWEL      112
sp|Q6UX41|BTNL8_HUMAN      GKDQPFMQMPQYQGRTKLVKDSIAEGRISLRLENITVLDAGLYGCRISSQSYYQKAIWEL      112
sp|Q6UXG8|BTNL9_HUMAN      QQELPGRQMPAFRNRTKLVKDDIAYGSVVLQLHSIIPSDKGTYGCRFHSDNFSGEALWEL      114
sp|A8MVZ5|BTNLA_HUMAN      GMDMDGEQKWQYRGRTTFMSDHVARGKAMVRSHRVTTFDNRTYCCRFKDGVKFGEATVQV      117
sp|Q96PL5|ERMAP_HUMAN      GKDQDEDLMPEYKGRTVLVRDA-QEGSVTLQILDVRLEDQGSYRCLIQVGNLSKEDTVIL      112
sp|Q16653|MOG_HUMAN        GKDQDGDQAPEYRGRTELLKDAIGEGKVTLRIRNVRFSDEGGFTCFFRDHSYQEEAAMEL      113
                             :        ::.    : .    *   :    :   :   : * :       :    :

sp|Q13410|BT1A1_HUMAN      KVAALGSDPHISMQVQENGEICLECTSVGWYPEPQVQWRTSKGEKFPST-SESRNPDEEG      172
sp|Q7KYR7|BT2A1_HUMAN      VVAGLGSKPLISMRGHEDGGIRLECISRGWYPKPLTVWRDPYGGVAPAL-KEVSMPDADG      171
sp|Q8WVV5|BT2A2_HUMAN      VVAGLGSKPLIEIKAQEDGSIWLECISGGWYPEPLTVWRDPYGEVVPAL-KEVSIADADG      171
sp|Q96KV6|BT2A3_HUMAN      VVAGLDSEPVIEMRDHEDGGIQLECISGGWYPKPLTVWRDPYGEVVPAL-KEVSTPDADS      170
sp|O00481|BT3A1_HUMAN      KVAALGSDLHVDVKGYKDGGIHLECRSTGWYPQPQIQWSNNKGENIPTV-EAPVVADGVG      171
```

```
sp|P78410|BT3A2_HUMAN      KVAALGSNLHVEVKGYEDGGIHLECRSTGWYPQPQIQWSNAKGENIPAV-EAPVVADGVG     171
sp|O00478|BT3A3_HUMAN      KVAALGSDLHIEVKGYEDGGIHLECRSTGWYPQPQIKWSDTKGENIPAV-EAPVVADGVG     171
sp|Q9UIR0|BTNL2_HUMAN      KVAGLGSAPSIHMEGPGESGVQLVCTARGWFPEPQVYWEDIRGEKLLAV-SEHRIQDKDG     171
sp|Q6UXE8|BTNL3_HUMAN      RVAALGSLPLISIVGYVDGGIQLLCLSSGWFPQPTAKWKGPQGQDLSSD-SRA-NADGYS     170
sp|Q6UX41|BTNL8_HUMAN      QVSALGSVPLISITGYVDRDIQLLCQSSGWFPRPTAKWKGPQGQDLSTD-SRT-NRDMHG     170
sp|Q6UXG8|BTNL9_HUMAN      EVAGLGSDPHLSLEGFKEGGIQLRLRSSGWYPKPKVQWRDHQGQCLPPE-FEAIVWDAQD     173
sp|A8MVZ5|BTNLA_HUMAN      QVAGLGREPRIQVTDQQD-GVRAECTSAGCFPKSWVERRDFRGQARPAV-TNLSASATTR     175
sp|Q96PL5|ERMAP_HUMAN      QVAAPSV-----------------------------------------------------     119
sp|Q16653|MOG_HUMAN        KVEDPF------------------------------YWVSPGVLVLLAVLPVLLLQITVG     143
                            *

sp|Q13410|BT1A1_HUMAN      LFTVAASVIIRDTSAKNVSCYIQNLLLGQEKKV-EISIPASSLPR--LTPWIVAVAVIL-     228
sp|Q7KYR7|BT2A1_HUMAN      LFMVTTAVIIRDKSVRNMSCSINNTLLGQKKES-VIFIPESFMPS--VSPCAVALPIIV-     227
sp|Q8WVV5|BT2A2_HUMAN      LFMVTTAVIIRDKYVRNVSCSVNNTLLGQEKET-VIFIPESFMPS--ASPWMVALAVILT     228
sp|Q96KV6|BT2A3_HUMAN      LFMVTTAVIIRDKSVRNVSCSINDTLLGQKKES-VIFIPESFMPS--RSPCVVILPVIM-     226
sp|O00481|BT3A1_HUMAN      LYAVAASVIMRGSSGEGVSCTIRSSLLGLEKTA-SISIADPFFRS--AQRWIAALAGTL-     227
sp|P78410|BT3A2_HUMAN      LYEVAASVIMRGGSGEGVSCIIRNSLLGLEKTA-SISIADPFFRS--AQPWIAALAGTL-     227
sp|O00478|BT3A3_HUMAN      LYAVAASVIMRGSSGGGVSCIIRNSLLGLEKTA-SISIADPFFRS--AQPWIAALAGTL-     227
sp|Q9UIR0|BTNL2_HUMAN      LFYAEATLVVRNASAESVSCLVHNPVLTEEKGS-VISLPEKLQTE--LASLKVNGPSQP-     227
sp|Q6UXE8|BTNL3_HUMAN      LYDVEISIIVQENAG-SILCSIHLAEQSHEVES-KVLIGETFFQP---SPWRLASI----     221
sp|Q6UX41|BTNL8_HUMAN      LFDVEISLTVQENAG-SISCSMRHAHLSREVES-RVQIGDTFFEP---ISWHLATK----     221
sp|Q6UXG8|BTNL9_HUMAN      LFSLETSVVVRAGALSNVSVSIQNLLLSQKKEL-VVQIADVFVPG--ASAWKSAFVATL-     229
sp|A8MVZ5|BTNLA_HUMAN      LWAVASSLTLWDRAVEGLSCSISSPLLPERRKVAESHLPATFSRSSQFTAWKAALPLIL-     234
sp|Q96PL5|ERMAP_HUMAN      -------------------------------------------GS--LSPSAVALAVIL-     133
sp|Q16653|MOG_HUMAN        LIFLCLQYRLRG----------------KLRAE-IENLHRTFDPH------------FLR     174


sp|Q13410|BT1A1_HUMAN      ------------MVLGLLTIGSIFFTWRLYNERPRER-----------------RNE---     256
sp|Q7KYR7|BT2A1_HUMAN      ------------VILMIPIAVCIYWINKLQKEKKILSGEKEFERETREIALKELEKERVQ     275
sp|Q8WVV5|BT2A2_HUMAN      ASPWMVSMTVILAVFIIFMAVSICCIKKLQREKKILSGEKKVEQEEKE------------     276
sp|Q96KV6|BT2A3_HUMAN      ------------IILMIPIAICIYWINNLQKEKKDSHL----------------------     252
sp|O00481|BT3A1_HUMAN      ------------PVLLLLLGGAGYFLWQQQEEKKTQFRKKKREQELREMAWSTMKQE---     272
sp|P78410|BT3A2_HUMAN      ------------PILLLLLAGASYFLWRQQKEITALSSEIESEQEMKEMGYAATERE---     272
sp|O00478|BT3A3_HUMAN      ------------PISLLLLAGASYFLWRQQKEKIALSRETEREREMKEMGYAATEQE---     272
sp|Q9UIR0|BTNL2_HUMAN      ----------ILVRVGEDIQ-LTCYLSP--------------------------------     244
sp|Q6UXE8|BTNL3_HUMAN      ------------LLGLLCGALCGVVMGM--------------------------------     237
sp|Q6UX41|BTNL8_HUMAN      ------------VLGILCCGLFFGIVGL--------------------------------     237
sp|Q6UXG8|BTNL9_HUMAN      ------------PLLLVLAALALGVLRKQRRSREKLRKQAEKR-----------------     260
sp|A8MVZ5|BTNLA_HUMAN      ------------VAMGLVIAGGICIFWKRQREKNKASLEEERE-----------------     265
```

```
sp|Q96PL5|ERMAP_HUMAN      ------------PVLVLLIMVCLCLIWKQRRAKEKLLYEHVTEVDNLLS---DHA---KE      175
sp|Q16653|MOG_HUMAN        VPCWKITLFVIVPVLGPLVALIICYNWLHRRLAGQFLEELRNPF----------------      218


sp|Q13410|BT1A1_HUMAN      ----FSSKERLLEELKW------------------KKATLHAVDVTLDPDTAHPHLFLYE      294
sp|Q7KYR7|BT2A1_HUMAN      KEEELQVKEKLQEELRW------------------RRTFLHAVDVVLDPDTAHPDLFLSE      317
sp|Q8WVV5|BT2A2_HUMAN      ------IAQQLQEELRW------------------RRTFLHAADVVLDPDTAHPELFLSE      312
sp|Q96KV6|BT2A3_HUMAN      -----MTFNLCLSLAGW------------------RRTFLHAANVVLDQDTGHPYLFVSE      289
sp|O00481|BT3A1_HUMAN      ----QSTRVKLLEELRWRSIQYASRGERHSAYNEWKKALFKPADVILDPKTANPILLVSE      328
sp|P78410|BT3A2_HUMAN      ----ISLRESLQEELKRKKIQYLTRGEESSSDTNKSA-----------------------      305
sp|O00478|BT3A3_HUMAN      ----ISLREKLQEELKWRKIQYMARGEKSLAYHEWKMALFKPADVILDPDTANAILLVSE      328
sp|Q9UIR0|BTNL2_HUMAN      ------KANAQSMEVRWDRS------------------HRYPAVHVYMDGDHVAGEQMAEY      281
sp|Q6UXE8|BTNL3_HUMAN      IIVFFKSKGKIQAELDWRR---------KHGQAELRDARKHAVEVTLDPETAHPKLCVSD      288
sp|Q6UX41|BTNL8_HUMAN      KIFFSKFQWKIQAELDWRR---------KHGQAELRDARKHAVEVTLDPETAHPKLCVSD      288
sp|Q6UXG8|BTNL9_HUMAN      QEKLTAELEKLQTELDWRR---------AEGQAEWRAAQKYAVDVTLDPASAHPSLEVSE      311
sp|A8MVZ5|BTNLA_HUMAN      ------------------------------------------------------------      265
sp|Q96PL5|ERMAP_HUMAN      KGKLHKAVKKLRSELKLKR---------AAANSGWRRARLHFVAVTLDPDTAHPKLILSE      226
sp|Q16653|MOG_HUMAN        ------------------------------------------------------------      218


sp|Q13410|BT1A1_HUMAN      DSKSVRLEDS-RQ------KLPEKTERFDSWPCVLGRETFTSGRHYWEVEVGDRTDWAIG      347
sp|Q7KYR7|BT2A1_HUMAN      DRRSVRRCPF-RH--L-GESVPDNPERFDSQPCVLGRESFASGKHYWEVEVENVIEWTVG      373
sp|Q8WVV5|BT2A2_HUMAN      DRRSVRRGPY-RQ------RVPDNPERFDSQPCVLGWESFASGKHYWEVEVENVMVWTVG      365
sp|Q96KV6|BT2A3_HUMAN      DKRSVTLDPS-R------ESIPGNPERFDSQLCVLGQESFASGKHYLEVDVENVIEWTVG      342
sp|O00481|BT3A1_HUMAN      DQRSVQRAKE-PQ------DLPDNPERFNWHYCVLGCESFISGRHYWEVEVGDRKEWHIG      381
sp|P78410|BT3A2_HUMAN      ------------------------------------------------------------      305
sp|O00478|BT3A3_HUMAN      DQRSVQRAEE-PR------DLPDNPERFEWRYCVLGCENFTSGRHYWEVEVGDRKEWHIG      381
sp|Q9UIR0|BTNL2_HUMAN      RGRTVLVSDAIDEGRLTLQILSARPSDDGQYRCLFE-----KDDVYQEASLDLK-VVSLG      335
sp|Q6UXE8|BTNL3_HUMAN      LK-TVTHRKA-PQ------EVPHSEKRFTRK-SVVASQGFQAGKHYWEVDVGQNVGWYVG      339
sp|Q6UX41|BTNL8_HUMAN      LK-TVTHRKA-PQ------EVPHSEKRFTRK-SVVASQSFQAGKHYWEVDGGHNKRWRVG      339
sp|Q6UXG8|BTNL9_HUMAN      DGKSVSSRGAPPG------PAPGHPQRFSEQTCALSLERFSAGRHYWEVHVGRRSRWFLG      365
sp|A8MVZ5|BTNLA_HUMAN      ------------------------------------------------------------      265
sp|Q96PL5|ERMAP_HUMAN      DQRCVRLGDR-RQ------PVPDNPQRFDFVVSILGSEYFTTGCHYWEVYVGDKTKWILG      279
sp|Q16653|MOG_HUMAN        ------------------------------------------------------------      218


sp|Q13410|BT1A1_HUMAN      VCRE-NVM--K-KGFDPMTPENGFWAVELY--------------------GNGYWALTPL      383
sp|Q7KYR7|BT2A1_HUMAN      VCRD-SVE--R-KGEVLLIPQNGFWTLEMH--------------------KGQYRAVSSP      409
sp|Q8WVV5|BT2A2_HUMAN      VCRH-SVE--R-KGEVLLIPQNGFWTLEMF--------------------GNQYRALSSP      401
```

```
sp|Q96KV6|BT2A3_HUMAN      ICRD-NVE--R-KWEVPLLPQNGFWTLEMH--------------------KRKYWALTSL      378
sp|O00481|BT3A1_HUMAN      VCSK-NVQ--R-KGWVKMTPENGFWTMGLT------------------D-GNKYRTLTEP      418
sp|P78410|BT3A2_HUMAN      ------------------------------------------------------------      305
sp|O00478|BT3A3_HUMAN      VCSK-NVE--RKKGWVKMTPENGYWTMGLT------------------D-GNKYRALTEP      419
sp|Q9UIR0|BTNL2_HUMAN      SSPLITVEGQEDGEMQPMCSSDGWFPQPHVPWRDMEGKTIPSSSQALTQGSHGLFHVQTL      395
sp|Q6UXE8|BTNL3_HUMAN      VCRD-DVD--RGKNNVTLSPNNGYWVLRLT------------------T-EHLYFTFNPH      377
sp|Q6UX41|BTNL8_HUMAN      VCRD-DVD--RRKEYVTLSPDHGYWVLRLN------------------G-EHLYFTLNPR      377
sp|Q6UXG8|BTNL9_HUMAN      ACLA-AVP--RA-GPARLSPAAGYWVLGLW------------------N-GCEYFVLAPH      402
sp|A8MVZ5|BTNLA_HUMAN      ------------------------------------------------------------      265
sp|Q96PL5|ERMAP_HUMAN      VCSE-SVS--R-KGKVTASPANGHWLLRQS------------------R-GNEYEALTSP      316
sp|Q16653|MOG_HUMAN        ------------------------------------------------------------      218


sp|Q13410|BT1A1_HUMAN      R--TPLPLAGPPRRVGIFLDYESGDISFYNMNDGSDIYTFSNVTFSGPLRPFFCLW----      437
sp|Q7KYR7|BT2A1_HUMAN      D--RILPLKESLCRVGVFLDYEAGDVSFYNMRDRSHIYTCPRSAFSVPVRPFFRLG----      463
sp|Q8WVV5|BT2A2_HUMAN      E--RILPLKESLCRVGVFLDYEAGDVSFYNMRDRSHIYTCPRSAFTVPVRPFFRLG----      455
sp|Q96KV6|BT2A3_HUMAN      K--WILSLEEPLCQVGIFLDYEAGDVSFYNMRDRSHIYTFPHSAFSVPVRPFFSLG----      432
sp|O00481|BT3A1_HUMAN      R--TNLKLPKPPKKVGVFLDYETGDISFYNAVDGSHIHTFLDVSFSEALYPVFRIL----      472
sp|P78410|BT3A2_HUMAN      ------------------------------------------------------------      305
sp|O00478|BT3A3_HUMAN      R--TNLKLPEPPRKVGIFLDYETGEISFYNATDGSHIYTFPHASFSEPLYPVFRIL----      473
sp|Q9UIR0|BTNL2_HUMAN      LRVTNISAVDVTCSISI---------PFL---GEEKIATFSLSGW---------------      428
sp|Q6UXE8|BTNL3_HUMAN      F--ISLPPSTPPTRVGVFLDYEGGTISFFNTNDQSLIYTLLTCQFEGLLRPYIQH-AMYD      434
sp|Q6UX41|BTNL8_HUMAN      F--ISVFPRTPPTKIGVFLDYECGTISFFNINDQSLIYT-LTCRFEGLLRPYIEY-PSYN      433
sp|Q6UXG8|BTNL9_HUMAN      R--VALTLRVPPRRLGVFLDYEAGELSFFNVSDGSHIFTFH-DTFSGALCAYFRPRAHDG      459
sp|A8MVZ5|BTNLA_HUMAN      ------------------------------------------------------------      265
sp|Q96PL5|ERMAP_HUMAN      Q--TSFRLKEPPRCVGIFLDYEAGVISFYNVTNKSHIFTFT-HNFSGPLRPFFEPCLHDG      373
sp|Q16653|MOG_HUMAN        ------------------------------------------------------------      218


sp|Q13410|BT1A1_HUMAN      SSGKKPLTICPIADGPERVTVIANAQDLSKE----------IPLSPMGEDSAPRDADTLH      487
sp|Q7KYR7|BT2A1_HUMAN      C-EDSPIFICPALTGANGVTVPEEGLTLHRV-GTHQSL----------------------      499
sp|Q8WVV5|BT2A2_HUMAN      S-DDSPIFICPALTGASGVMVPEEGLKLHRV-GTHQSL----------------------      491
sp|Q96KV6|BT2A3_HUMAN      S-YDSQILICSAFTGASGVTVPEEGWTLHRA-GTHHSPQNQFPSLTAMETSPGHLSSHCT      490
sp|O00481|BT3A1_HUMAN      TLEPTALTICPA------------------------------------------------      484
sp|P78410|BT3A2_HUMAN      ------------------------------------------------------------      305
sp|O00478|BT3A3_HUMAN      TLEPTALTICPIPKEVESSPDPDLVPDHSLE----------TPLTPGLANESGEPQAEVT      523
sp|Q9UIR0|BTNL2_HUMAN      ------------------------------------------------------------      428
sp|Q6UXE8|BTNL3_HUMAN      EEKGTPIFICPVSWG---------------------------------------------      449
sp|Q6UX41|BTNL8_HUMAN      EQNGTPIVICPVTQESEKEASWQRASAIPETSNSESSSQATTPFLPRG------------      481
```

```
sp|Q6UXG8|BTNL9_HUMAN      GEHPDPLTICPLPVRGT---------GVPEENDSDTWL---QPYEPAD------------    495
sp|A8MVZ5|BTNLA_HUMAN      ------------------------------------------------------------    265
sp|Q96PL5|ERMAP_HUMAN      GKNTAPLVICSELHKSEESIVPRPEGKGHAN--GDVSLKVNSSLLPPKAPEL-------K    424
sp|Q16653|MOG_HUMAN        ------------------------------------------------------------    218


sp|Q13410|BT1A1_HUMAN      SKLIPTQPSQGAP-----------------------------------------------    500
sp|Q7KYR7|BT2A1_HUMAN      ------------------------------------------------------------    499
sp|Q8WVV5|BT2A2_HUMAN      ------------------------------------------------------------    491
sp|Q96KV6|BT2A3_HUMAN      MPLVEDTPSS---PLVTQENIFQLPLSHPLQTS-APVHLLIRCGFSSSFGCNYGMESRHR    546
sp|O00481|BT3A1_HUMAN      ------------------------------------------------------------    484
sp|P78410|BT3A2_HUMAN      ------------------------------------------------------------    305
sp|O00478|BT3A3_HUMAN      SLLLPAHPGAEVSPSATTNQ------NHKLQAR-TEAL--------------Y-------    555
sp|Q9UIR0|BTNL2_HUMAN      ------------------------------------------------------------    428
sp|Q6UXE8|BTNL3_HUMAN      ------------------------------------------------------------    449
sp|Q6UX41|BTNL8_HUMAN      -------EM---------------------------------------------------    483
sp|Q6UXG8|BTNL9_HUMAN      -------PALDWW-----------------------------------------------    501
sp|A8MVZ5|BTNLA_HUMAN      ------------------------------------------------------------    265
sp|Q96PL5|ERMAP_HUMAN      DIILSLPPDLG--P--------------ALQELKAPSF----------------------    446
sp|Q16653|MOG_HUMAN        ------------------------------------------------------------    218


sp|Q13410|BT1A1_HUMAN      -------------    500
sp|Q7KYR7|BT2A1_HUMAN      -------------    499
sp|Q8WVV5|BT2A2_HUMAN      -------------    491
sp|Q96KV6|BT2A3_HUMAN      ELVVPQLPARKKV    559
sp|O00481|BT3A1_HUMAN      -------------    484
sp|P78410|BT3A2_HUMAN      -------------    305
sp|O00478|BT3A3_HUMAN      -------------    555
sp|Q9UIR0|BTNL2_HUMAN      -------------    428
sp|Q6UXE8|BTNL3_HUMAN      -------------    449
sp|Q6UX41|BTNL8_HUMAN      -------------    483
sp|Q6UXG8|BTNL9_HUMAN      -------------    501
sp|A8MVZ5|BTNLA_HUMAN      -------------    265
sp|Q96PL5|ERMAP_HUMAN      -------------    446
sp|Q16653|MOG_HUMAN        -------------    218
```

Figure S2. Percent Identity Matrix created by Clustal 2.1 for human butyrophilin family members

sp|Q13410|BT1A1_HUMAN	100.00	49.14	50.33	44.99	50.89	47.78	49.10	32.82	39.07	35.46	40.69	36.51	42.05	35.53
sp|Q7KYR7|BT2A1_HUMAN	49.14	100.00	84.52	78.63	44.18	42.51	45.34	31.55	39.72	35.46	39.53	38.52	40.92	29.56
sp|Q8WVV5|BT2A2_HUMAN	50.33	84.52	100.00	74.03	46.36	44.57	46.40	31.63	38.64	35.04	38.10	37.35	43.27	30.56
sp|Q96KV6|BT2A3_HUMAN	44.99	78.63	74.03	100.00	41.63	39.10	40.47	30.59	36.15	31.81	35.19	37.45	36.63	28.57
sp|O00481|BT3A1_HUMAN	50.89	44.18	46.36	41.63	100.00	82.30	85.74	26.65	38.44	36.70	41.18	35.02	37.63	32.02
sp|P78410|BT3A2_HUMAN	47.78	42.51	44.57	39.10	82.30	100.00	89.84	33.59	36.02	32.18	37.94	35.41	28.35	33.00
sp|O00478|BT3A3_HUMAN	49.10	45.34	46.40	40.47	85.74	89.84	100.00	28.10	38.32	34.84	41.20	37.35	37.97	32.51
sp|Q9UIR0|BTNL2_HUMAN	32.82	31.55	31.63	30.59	26.65	33.59	28.10	100.00	24.61	25.19	25.95	31.95	22.37	32.98
sp|Q6UXE8|BTNL3_HUMAN	39.07	39.72	38.64	36.15	38.44	36.02	38.32	24.61	100.00	74.33	43.18	29.66	36.36	32.61
sp|Q6UX41|BTNL8_HUMAN	35.46	35.46	35.04	31.81	36.70	32.18	34.84	25.19	74.33	100.00	40.72	29.24	34.90	34.24
sp|Q6UXG8|BTNL9_HUMAN	40.69	39.53	38.10	35.19	41.18	37.94	41.20	25.95	43.18	40.72	100.00	29.34	37.63	31.03
sp|A8MVZ5|BTNLA_HUMAN	36.51	38.52	37.35	37.45	35.02	35.41	37.35	31.95	29.66	29.24	29.34	100.00	31.06	30.54
sp|Q96PL5|ERMAP_HUMAN	42.05	40.92	43.27	36.63	37.63	28.35	37.97	22.37	36.36	34.90	37.63	31.06	100.00	38.41
sp|Q16653|MOG_HUMAN	35.53	29.56	30.56	28.57	32.02	33.00	32.51	32.98	32.61	34.24	31.03	30.54	38.41	100.00

Figure S3. Phylogenetic Tree created for the human butyrophilin family members by the CLUSTAL Omega (1.2.4) algorithm. This is a Neighbor-joining tree without distance corrections.

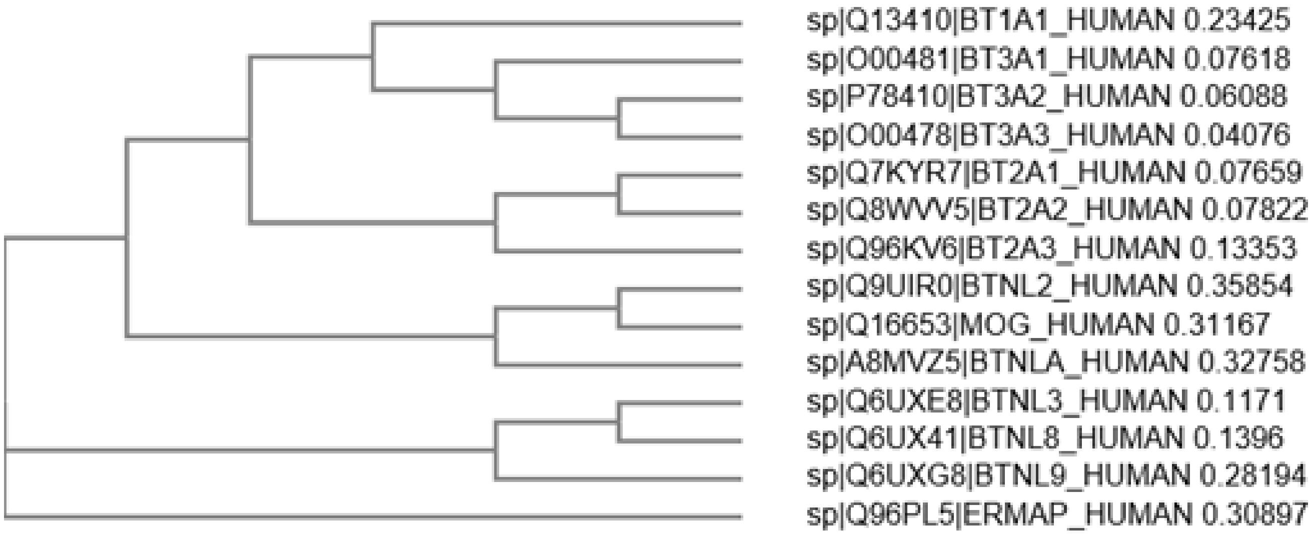

Figure S4. Analysis of the interactivity of the human BTN2A1 (**A**), BTN2A2 (**B**), BTN3A1 (**C**), BTN3A2 (**D**), BTN3A2 (**E**), BTNL2 (**F**), BTNL8 (**G**), BTNL9 (**H**), and MOG (**I**) by STRING platform (http://string-db.org/cgi/). STRING produces the network of predicted associations for a particular protein and its interactome. The network nodes are proteins, whereas the edges represent the predicted or known functional associations. There are seven types of evidence used in predicting the associations which are indicated in the resulting network by the differently colored lines, where a red line indicates the presence of fusion evidence; a green line - neighborhood evidence; a blue line – co-occurrence evidence; a purple line - experimental evidence; a yellow line – text mining evidence; a light blue line - database evidence; a black line – co-expression evidence.

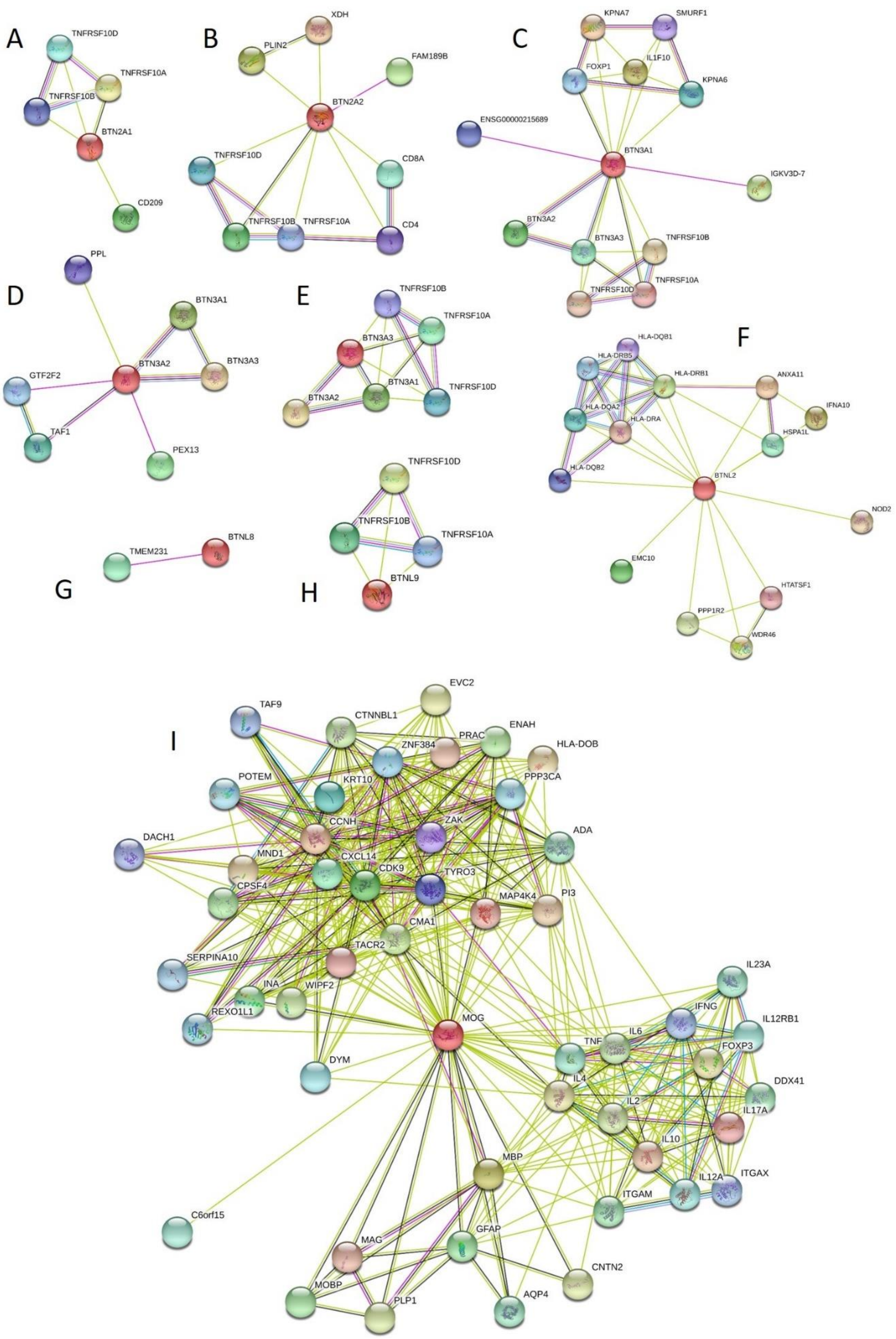
A
TNFRSF10D
TNFRSF10A
TNFRSF10B
BTN2A1
CD209
B
XDH
PLIN2
FAM189B
BTN2A2
TNFRSF10D
CD8A
TNFRSF10B
TNFRSF10A
CD4
C
KPNA7
SMURF1
FOXP1
IL1F10
KPNA6
ENSG00000215689
BTN3A1
IGKV3D-7
BTN3A2
BTN3A3
TNFRSF10B
TNFRSF10D
TNFRSF10A
D
PPL
BTN3A1
GTF2F2
BTN3A2
BTN3A3
TAF1
PEX13
E
TNFRSF10B
TNFRSF10A
BTN3A3
BTN3A1
TNFRSF10D
BTN3A2
F
HLA-DQB1
HLA-DRB5
HLA-DRB1
ANXA11
IFNA10
HLA-DQA2
HLA-DRA
HSPA1L
HLA-DQB2
BTNL2
NOD2
EMC10
HTATSF1
PPP1R2
WDR46
G
TMEM231
BTNL8
H
TNFRSF10D
TNFRSF10B
TNFRSF10A
BTNL9
I
EVC2
TAF9
CTNNBL1
ENAH
PRAC
ZNF384
HLA-DOB
POTEM
KRT10
PPP3CA
CCNH
ZAK
ADA
DACH1
MND1
CXCL14
CDK9
TYRO3
CPSF4
MAP4K4
PI3
CMA1
TACR2
SERPINA10
INA
WIPF2
REXO1L1
MOG
DYM
IL23A
IFNG
IL12RB1
TNF
IL6
FOXP3
IL4
DDX41
IL2
IL17A
IL10
MBP
IL12A
ITGAX
ITGAM
C6orf15
MAG
GFAP
CNTN2
MOBP
PLP1
AQP4
